# First Comparative Analysis of *Clostridium septicum* Genomes Provides Insights Into the Taxonomy, Species Genetic Diversity, and Virulence Related to Gas Gangrene

**DOI:** 10.3389/fmicb.2021.771945

**Published:** 2021-12-09

**Authors:** Prasad Thomas, Mostafa Y. Abdel-Glil, Anbazhagan Subbaiyan, Anne Busch, Inga Eichhorn, Lothar H. Wieler, Heinrich Neubauer, Mathias Pletz, Christian Seyboldt

**Affiliations:** ^1^Institute of Bacterial Infections and Zoonoses, Friedrich-Loeffler-Institut, Jena, Germany; ^2^Division of Bacteriology and Mycology, ICAR-Indian Veterinary Research Institute, Izatnagar, India; ^3^Institute for Infectious Diseases and Infection Control, Jena University Hospital – Friedrich Schiller University, Jena, Germany; ^4^Department of Pathology, Faculty of Veterinary Medicine, Zagazig University, Zagazig, Egypt; ^5^Department of Anaesthesiology and Intensive Care Medicine, University Hospital Jena, Jena, Germany; ^6^Department of Veterinary Medicine, Institute of Microbiology and Epizootics, Freie Universität Berlin, Berlin, Germany; ^7^Robert Koch Institute, Berlin, Germany

**Keywords:** *Clostridium septicum*, comparative analysis, genome, gas gangrene, DSM 7534^T^, virulence factors

## Abstract

*Clostridium septicum* is a Gram-positive, toxin-producing, and spore-forming bacterium that is recognized, together with *C. perfringens*, as the most important etiologic agent of progressive gas gangrene. *Clostridium septicum* infections are almost always fatal in humans and animals. Despite its clinical and agricultural relevance, there is currently limited knowledge of the diversity and genome structure of *C. septicum*. This study presents the complete genome sequence of *C. septicum* DSM 7534^T^ type strain as well as the first comparative analysis of five *C. septicum* genomes. The taxonomy of *C. septicum*, as revealed by 16S rRNA analysis as well as by genomic wide indices such as protein-based phylogeny, average nucleotide identity, and digital DNA–DNA hybridization indicates a stable clade. The composition and presence of prophages, CRISPR elements and accessory genetic material was variable in the investigated genomes. This is in contrast to the limited genetic variability described for the phylogenetically and phenotypically related species *Clostridium chauvoei*. The restriction-modification (RM) systems between two *C. septicum* genomes were heterogeneous for the RM types they encoded. *C. septicum* has an open pangenome with 2,311 genes representing the core genes and 1,429 accessory genes. The core genome SNP divergence between genome pairs varied up to 4,886 pairwise SNPs. A vast arsenal of potential virulence genes was detected in the genomes studied. Sequence analysis of these genes revealed that sialidase, hemolysin, and collagenase genes are conserved compared to the α-toxin and hyaluronidase genes. In addition, a conserved gene found in all *C. septicum* genomes was predicted to encode a leucocidin homolog (beta-channel forming cytolysin) similar (71.10% protein identity) to *Clostridium chauvoei* toxin A (CctA), which is a potent toxin. In conclusion, our results provide first, valuable insights into strain relatedness and genomic plasticity of *C. septicum* and contribute to our understanding of the virulence mechanisms of this important human and animal pathogen.

## Introduction

*Clostridium septicum*, the first anaerobic pathogen described (MacLennan, 1962) is the major causative agent of a pathologic fatal condition in animals and humans named as malignant edema or gas gangrene that may also involve other *Clostridia* species such as *C. perfringens* type A, *C. novyi*, *C. chauvoei*, and *C. sordellii* (MacLennan, 1962). Malignant edema in animals arises predominantly from exogenous wound contamination. This is in contrast to blackleg disease that is non-traumatic endogenous infection mainly reported in cattle and caused by *C. chauvoei* (Silva et al., 2016). *Clostridium septicum* induced malignant edema has a broad host range with disease reported in ruminants, horses, pigs, elephants, and birds (Sasaki et al., 2001; Odani et al., 2009; Rahman et al., 2009; Almeida E Macêdo et al., 2013). Animals of all ages can be affected and the disease occurs worldwide. Rare outbreaks in animals due to injection of contaminated vaccines or other medical interventions have also been reported (Morris et al., 2002). *Clostridium septicum* infections leading to vulvovaginitis and metritis after parturition (post parturient malignant oedema) in cattle (Odani et al., 2009; Junior et al., 2020) and necrotizing abomasitis (braxy) in lambs and calves were reported (Schamber et al., 1986; Glenn Songer, 2009). In humans, *C. septicum* was reported to cause atraumatic gas gangrene in immune-compromised patients (Barnes et al., 2004). Association of *C. septicum*-mediated myonecrosis in human patients with colonic (Nanjappa et al., 2015) or hematologic malignancy resulting in high mortality rates (79%) was also described (Kornbluth et al., 1989; Powell et al., 2008). In cases of fatal gas gangrene with multiorgan involvement and in a rare aortitis in human patients with colonic malignancy, *C. septicum* was detected in blood and tissue cultures (Seder et al., 2009; Curtis-Martínez and Sánchez-Guillén, 2019; Kousa et al., 2020). Table 1 lists selected human and animal disease reports and conditions primarily associated with *C. septicum*, indicating its relevance in the medical and veterinary settings. Despite this relevance, current information on genomic features and diversity of the species is limited to strain P1044, isolated from human intestine, whose genome is represented by a draft sequence and briefly described in a genome announcement (Benamar et al., 2016). Genetic information on the diversity of the pathogen is also limited, with few studies focusing on the genetic diversity of the α-toxin gene (Amimoto et al., 2006). Multi-locus sequence typing (MLST) analysis of poultry *C. septicum* strains associated with gangrenous dermatitis revealed a high conservation among selected MLST genes (Neumann and Rehberger, 2009).

**Table 1:** List of selected disease reports and conditions in humans and animals that are mainly linked to *C. septicum*.

Diseases/condition	Host(s)	Cardinal features and prognosis	References
Gas gangrene or malignant oedema	Ruminants, horses and other animals	Fever, subcutaneous oedema and emphysema. Dark red discoloration and petechiae in affected areas. Death following toxaemia and systemic shock.	Silva et al., 2016; Gazioglu et al., 2018; Junior et al., 2020
Post parturient malignant oedema	Cattle	Fever, necrotizing vulvovaginitis and metritis. Hemorrhagic perineal, multifocal necrosis and ulceration in vulvar and vaginal mucosae. Death usually within 24 h after onset of clinical signs.	Odani et al., 2009; Junior et al., 2020
Gangrenous dermatitis	Broiler chickens and turkeys	High fever, leg weakness and ataxia. Subcutaneous oedema in lower abdomen and inner thighs, dark-red to purple discoloration of skin Acute mortality in birds.	Willoughby et al., 1996; Shivaprasad, 2016; Gornatti-Churria et al., 2018
Necrotizing abomasitis (“braxy”)	Lambs and calves	Sudden onset, bloating and fever Edema, necrosis, congestion in abomasal wall and Blood tinged abomasal contents. Death usually occurs before clinical signs are noticed.	Schamber et al., 1986; Glenn Songer and Miskimins, 2005; Glenn Songer, 2009
Gas gangrene (Atraumatic myonecrosis)	Human	Mostly associated with malignancy. Fever, pain and tachycardia. Discoloration, crepitus of affected area and necrotizing fasciitis. Sepsis, failure of treatment leads to mortality.	Kornbluth et al., 1989; Smith-Slatas et al., 2006; Srivastava et al., 2017
Aortitis, aortic dissection and aortic aneurysm	Humans	Mostly associated with malignancy. Fever, chest pain, presence of periaortic gas and leukocytosis. Death may occur without adequate treatments	Seder et al., 2009; Annapureddy et al., 2012; Eplinius and Hädrich, 2014; Ito et al., 2017

Furthermore, the toxin repertoire of *C. septicum* has not been studied in detail. *C. septicum* is known to produce four types of toxins: alpha, beta, gamma, and delta toxin (Moussa, 1958; Ballard et al., 1992; Amimoto et al., 2006). The best studied of these is α-toxin, a potent pore-forming toxin that is released extracellularly (Bernheimer, 1944; Kennedy et al., 2005, 2009). Studies have shown the importance of α-toxin in virulence and development of myonecrosis (Kennedy et al., 2005; Hickey et al., 2008), and a recent study has shown that α-toxin could modulate the host innate immune response (Chakravorty et al., 2015).

The aim of the current study was to (1) generate a high-quality complete genome sequence of the type strain (DSM 7534^T^ = ATCC 12464) using a combination of short and long reads and (2) perform a comparative genome analysis of *C. septicum* strains, using genome data sets from public databases. Analysis of the genomes with respect to species taxonomy, repeat regions, prophage elements and virulence factors was carried out for all genomes. In addition, methylation motifs (one genome) and restriction-modification (RM) systems of two finished *C. septicum* genomes were investigated and described.

## Materials and Methods

### Genome Sequencing and Assembly (DSM 7534^T^)

*C. septicum* type strain DSM 7534^T^ (ATCC 12464, CIP 61.10, NCIMB 547, and NCTC 547) was sequenced. Genomic DNA was extracted using Qiagen Genomic-tip 100/Q (Qiagen, Germany) with minor modifications as the washing step was repeated five times and the DNA was incubated at 37°C until dissolved. DNA quality was examined by using Qubit 2.0 fluorometer (Life Technologies, Germany) and species confirmation was made with PCR (Sasaki et al., 2000). Genome sequencing was carried out by Pacific Biosciences (PacBio) sequencing using PacBio RSII sequencer at GATC Biotech (Germany). Additional sequencing for the same strain was carried out using MiSeq™ System (Illumina, United States) paired-end sequencing technology (2 × 300-bp) at the Institute of Microbiology and Epizootics (IMT), Freie Universität Berlin. Genome assembly was carried out using Unicycler, run under the bold mode (Wick et al., 2017). The unfinished contigs generated by Unicycler were circularized using Circlator (Hunt et al., 2015).

### Retrieval and Processing of Publicly Available Sequence Data

For the analysis, genomes (whole-genome assemblies and raw sequencing reads) available in NCBI for *C. septicum* (taxonomy number 1504) were downloaded (March 2021). They comprise genome assemblies of strain VAT12 (accession NZ_CP034358) isolated in 2012 in Virginia, the United States from a wild turkey and strain P1044 (accession NZ_FLTT00000000.1) isolated from a human stool in Marseille, France. A duplicate genome of strain P1044 was available under the accession no. NZ_CABMIZ000000000.1 (genome MGYG-HGUT-02373; Almeida et al., 2021). In addition, the sequence read archive database of NCBI included raw sequence data for only three *C. septicum* strains. Strain RVDL ALI_Clost_septicum_01 (hereafter RVDL_ALI; accession SRR10484857) sequenced using Illumina MiSeq was isolated in 2017 from a calf in Saudi Arabia. Strains DRS014147 (accession, DRR016039) and ERS2884028 (accession ERR3283734) were sequenced using Illumina HiSeq™ system. Epidemiological data were not available for the latter two strains. Initial taxonomy analysis using Kranken2 (Wood et al., 2019) classified the genome ERS2884028 to the species *Streptococcus pneumonia*, hence it was excluded. Paired-end Illumina reads were assembled with Shovill v1.0.4 using the option “-trim” enabling adapter and quality trimming using Trimmomatic (Wood et al., 2019).^1^ Assembly statistics were reported using QUAST v5.0.2 (Gurevich et al., 2013). In total, five genome sequences of *C. septicum* strains representing different regions and hosts were included in our analysis. Genome annotations were carried out using Prokka v1.14.6 (Seemann, 2014) in Galaxy server (Afgan et al., 2018).

### Taxonomic Classification

Taxonomic classification of *C. septicum* genomes was done first using the classical 16S rRNA gene sequence. The rRNA genes were predicted using barrnap v0.93 with default settings.^2^ In the case of genomes with multiple hits for the 16S rRNA gene, only one representative sequence (1.4-kb) was used and for partial 16S rRNA representations, the sequence with the highest alignment coverage was chosen. The 16S rRNA from all genomes was aligned using MAFFT v7.221 (Katoh et al., 2002). IQ-TREE v1.5.5.3 was used for maximum-likelihood (ML) phylogenetic analysis (Katoh et al., 2002) using ultrafast bootstrap approximation approach (UFBoot) and with 1,000 bootstraps (Minh et al., 2013; Nguyen et al., 2015). Tree visualization was done using iTOL v6 (Letunic and Bork, 2019).

For genome-wide approaches, we used PhyloPhlAn v0.43 to investigate species taxonomy by constructing a phylogenetic tree based on conserved ubiquitous proteins in bacteria (Segata et al., 2013). For that, an ML phylogeny was performed with RAxML v8.2.12 using the PROTCATLG model and 100 bootstrap replicates (Stamatakis, 2014). We also estimated the whole-genome-based average nucleotide identity (ANI) with pyani v0.2.3 (module: ANIm; Pritchard et al., 2016). For all the above-mentioned taxonomy analyses, representative genomes of all species of the genus cluster *Clostridium sensu stricto* were included for comparison (Supplementary Table S1). In addition, we calculated the genome-to-genome distances by using an *in silico* DNA–DNA hybridization (isDDH) approach as implemented in Genome-to-Genome Distance Calculator (GGDC) 2.1 webserver (Meier-Kolthoff et al., 2013; Jain et al., 2018). For that, the query genome assembly files were subjected to local alignments with BLAST+ tool (Camacho et al., 2009) against the reference *C. septicum* genome DSM 7534^T^ and estimates independent of genome lengths were used for distance calculations as recommended for draft genomes (Formula 2; Auch et al., 2010). For the isDDH analysis, *C. chauvoei* genome sequences (accessions NZ_CP018624 and NZ_LT799839.1) were kept as outgroups.

### Restriction-Modification Systems and Methylation Analysis

Single-molecule real-time (SMRT) sequencing by PacBio RSII allows the detection of base modifications in the genomes (Clark et al., 2012; Meier-Kolthoff et al., 2014). The RM systems information for *C. septicum* DSM 7534^T^ and VAT12 strains were retrieved from the REBASE website (Schadt et al., 2013).^3^ The methylated bases and methylation-associated motifs (DSM 7534^T^) were identified using the RS_Modification_and_Motif_analysis.1 tool in SMRT portal v2.3.0.

### Genome Comparison

Schematic representations of complete circular chromosomes and plasmids were generated using the CGView Server (Grant and Stothard, 2008). Comparative genomics involved five genome datasets. These genomes were investigated for CRISPR (clustered regularly interspaced short palindromic repeats) loci using the CRISPRDetect v2.4 (Biswas et al., 2016) with a minimum number of repeats > = 5. The CRISPR spacers were visualized using CRISPRStudio (Dion et al., 2018) in the Galaxy server (Afgan et al., 2018). Prophage elements were predicted using PHASTER (Arndt et al., 2016). Orthologous clustering of genes (pangenome analysis) was carried out using Prokka v1.14.6 (Seemann, 2014) annotated genomes with Panaroo pipeline v1.2.7 (Tonkin-Hill et al., 2020). Curves for the core and pangenome were calculated and plotted using PanGP v1.0.1 (Zhao et al., 2014). The pangenome plots were created also using GView Server with BLAST options (expect value 1-e 10, alignment length 100 and percent identity 80) and the GView v7.1 (Stothard et al., 2019).^4^ Prediction of antimicrobial resistance (AMR) genes was perfromed using ABRicate v1.0.1 and ResFinder (Zankari et al., 2012), CARD (McArthur et al., 2013) and AGR-Annot (Gupta et al., 2014) databases.^5^ Functional annotation of the core and accessory genes was carried out for Clusters of Orthologous Groups (COGs) using the eggnog-mapper v2 (Huerta-Cepas et al., 2017) based on eggNOG 5.0 orthology data (Huerta-Cepas et al., 2019) specifying the genus *Clostridia*. Additonally, the metabolic pathways were assessed using the BlastKOALA annotation server that use Kyoto Encyclopedia of Genes and Genomes (KEGG) Tools for functional characterization of protein coding sequences (Kanehisa et al., 2016). Positional homology multiple genome alignments of genome sequences were done using progressiveMauve (Darling et al., 2010). Core genome alignment generated using Panaroo was used for an ML phylogenetic analysis in IQ-TREE v1.5.5.3 using UFBoot as mentioned earlier (Minh et al., 2013; Nguyen et al., 2015). Tree visualization was done using iTOL v6 (Letunic and Bork, 2019). Pairwise core genome SNP variations between strains were determined using snp-dists v0.6.3.^6^

### Virulence Factors

Primary virulence factors for *C. septicum* were identified with BLASTP v2.9.0+ (Camacho et al., 2009) using a custom database constructed based on the virulence factor database (Liu et al., 2019) and putative virulence factors previously reported in the *C. chauvoei* type strain DSM 7528^T^ (NZ_CP018624). From the BLAST output results, we only kept BLAST hits with e-value of less than 1e-20, protein sequence identity more than 40%, coverage more than 70%, and total query gene length more than 90% of the total reference gene length. Nucleotide and protein alignments were performed for identified virulence factors using MAFFT v7.221 (Katoh et al., 2002). The amino acid (aa) variations between strains were visualized from alignments using Geneious Prime® 2019.2.3 (Kearse et al., 2012). Phylogenetic analysis was carried using IQ-TREE as mentioned above.

For the identified putative novel toxin homolog (beta-channel forming cytolysin: Locus tag-CP523_11160), feature and genetic structure predictions of signal peptide and Leukocidin/Hemolysin domain were carried out using SignalP v. 5.0 (Almagro Armenteros et al., 2019) and HMMER web server (Potter et al., 2018) searches to Pfam database (Mistry et al., 2021), respectively. An ML tree involving putative *C. septicum* toxin homolog with representative toxins from related species were carried out from protein alignment. Comparison of B cell epitopes of *C. septicum* cytolysin and CctA was carried out using ABCpred webserver (Saha and Raghava, 2006) with the settings threshold score 0.75; window length 20; overlapping filter –ON.

## Results

### Genome Features

**Figure 1:**
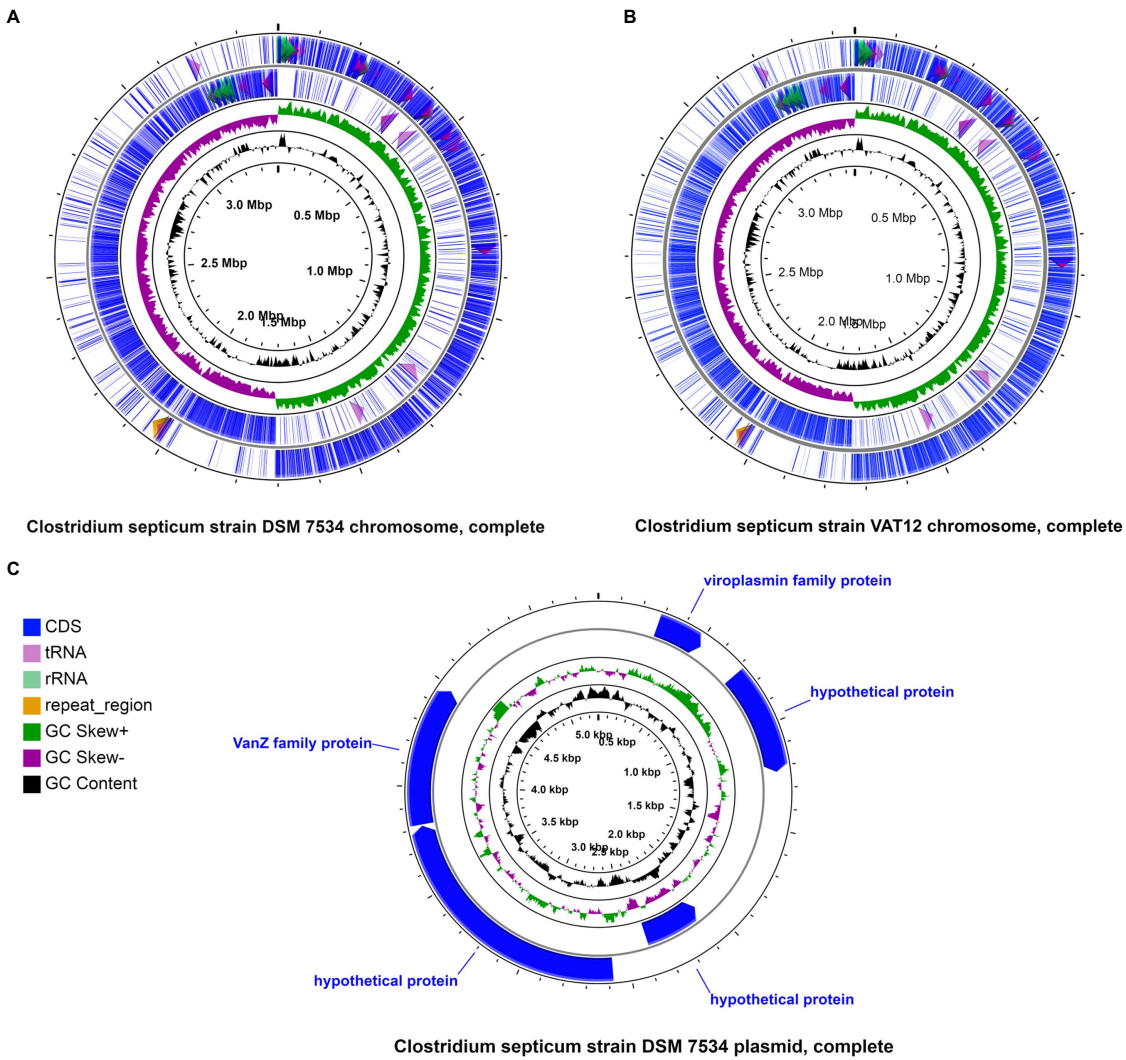
Schematic representation of circular chromosomes and plasmid. Circular chromosomes of DSM 7534^T^ **(A)** and VAT12 **(B)** strains and the circular plasmid of DSM 7534^T^ **(C)** strain are shown highlighting CDS, tRNA, rRNA, CRISPR elements, GC content and GC Skew.

The finished genome of the *C. septicum* type strain DSM 7534^T^ was determined, revealing a circular 3.3-Mb chromosome (NZ_CP023671) and a 5.2-kb plasmid (NZ_CP023672.1; Figure 1). Strain VAT 12 was also represented by a completed genome containing a circular 3.45-Mb chromosome (Figure 1). The other three genomes were in draft form (Table 2). Strains DRS014147 and RVDL_ALI had 125 and 578 contigs, representing 3.26 and 2.88-Mb genomes, respectively. The genomes of strain P1044 was represented by 79 contigs with a total genome size of 3.29-Mb. The overall genome size and GC composition of all assembled genomes were in similar ranges, except for strain RVDL_ALI. This strain had a smaller genome with a relatively higher GC content (28.2% compared with 27.5–27.9% for the other strains) and a lower number of predicted protein-coding genes (2,581 compared with 3,028–3,376 for the other genomes). These results could indicate poor sequencing or assembly of the genome which is also reflected in the genome high fragmentation: 578 contigs compared to less than 125 contigs for the other genomes (Table 2).

**Table 2:** Genome assembly, annotation, and metadata summary.

	Finished genomes		Genomes in a draft form		
	DSM 7534 T	VAT 12	P1044 (MGYG-HGUT-02373)	DRS014147	RVDL_ALI
n. contigs	2	1	79	125	578
N50 (bp)	3,454,144	3,454,144	71,267	60,346	8,676
Largest contig (bp)	3,399,422	3,454,144	213,922	141,085	69,475
Genome size (kb)	Chromosome: 3399 Plasmid: 5	Chromosome: 3454	3,298	3,266	2,887
GC content (percentage)	27.90%	27.9%	27.50%	27.50%	28.22%
	24.70%	-	28%		-
n. genes	3,231	3,422	3,143	3,084	2,648
n. rRNA	33	33	14	4	7
n. tRNA	87	79	81	52	60
n. CDS	3,111	3,376	3,048	3,028	2,581
Antimicrobial resistance genes^*^	-	-	-	-	tetA(P) & tetB(P)
Accession	NZ_CP023671 & NZ_CP023672.1	NZ_CP034358	NZ_FLTT00000000.1/ NZ_CABMIZ000000000.1	DRR016039	SRR10484857
Host	Unknown	Wild turkey	Human	Unknown	Calf
Country	Unknown	USA	France	Unknown	Saudi Arabia

* *Resistance genes were predicted using CARD, Resfinder and ARG-ANNOT databases with BLAST nucleotide identity and coverage cutoff above 80%. The identified genes in the genome RVDL_ALI encoding for Tet P which confers resistance to tetracycline*.

### Species Taxonomy

Phylogenetic analysis of the 16S rRNA gene revealed the clustering of *C. septicum* genomes in a monophyletic lineage. Nevertheless, grouping was observed between individual genomes of the species, for example, the genomes VAT 12 and P1044 formed a subgroup that was distinct from the other subgroup of the type strain (DSM 7534^T^) and the genomes of strains DRS014147 and RVDL_ALI (Supplementary Figure S1). The closely related species from *Clostridium sensu stricto* was *C. chauvoei* with 100% bootstrap support.

**Figure 2:**
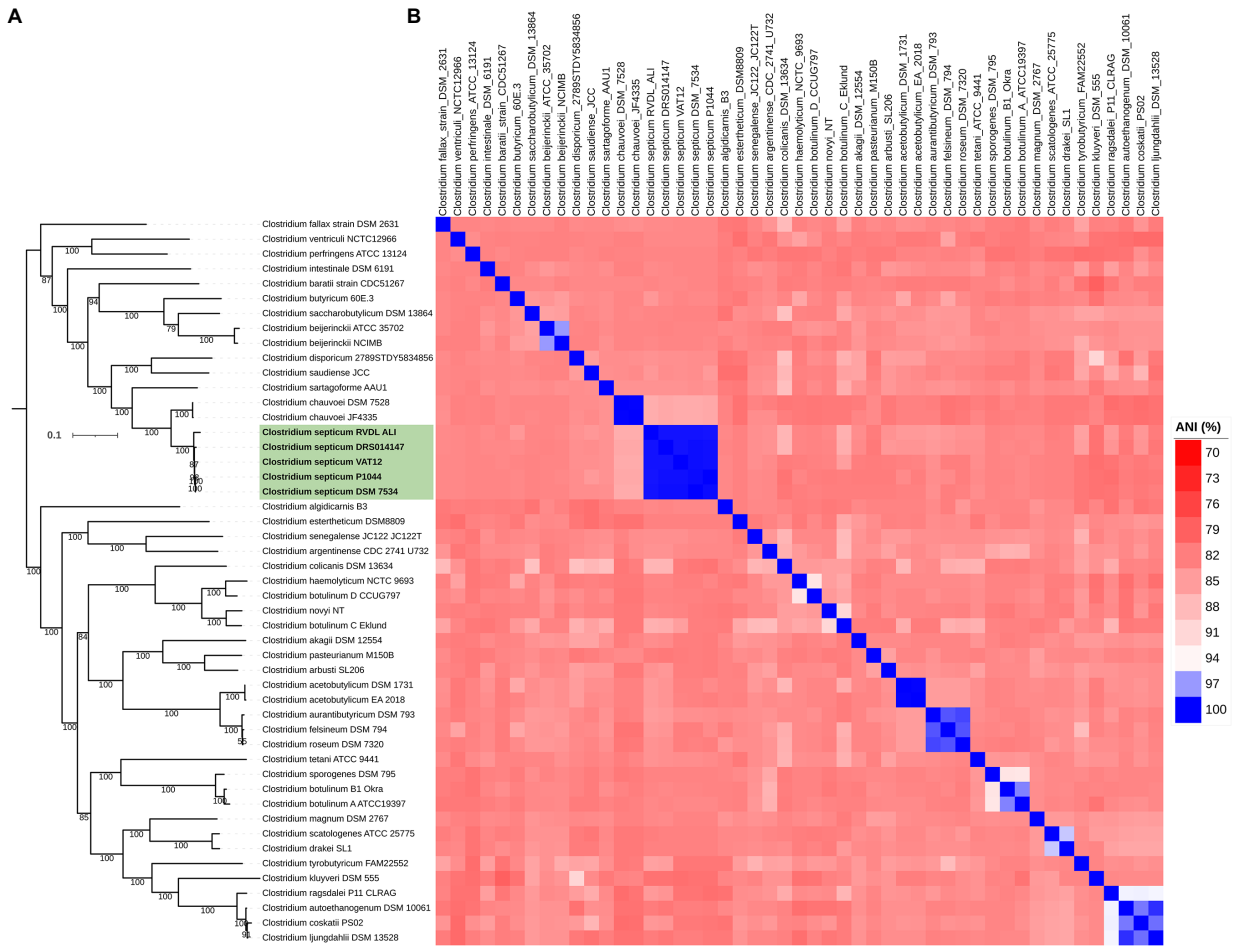
Taxonomic classification of *C. septicum* based on genome-wide approaches. Representative genomes of the *Clostridium* genus cluster 1 (*Clostridium sensu stricto*) were involved in the analysis. **(A)** Maximum likelihood tree based on PhyloPhlAn. **(B)** Pairwise average nucleotide identity (ANI) between the genomes.

The results of 16S rRNA analysis were further upheld by analysis of the whole genome, in which the phylogenetic tree based on the concatenated protein alignment of 400 universal bacterial genes placed the genomes of *C. septicum* in a separate group with close association to the species *C. chauvoei* (Figure 2A). ANI values between pairs of the five *C. septicum* genome datasets were more than 99% indicating that all five different genomes met the single species criteria. Pairwise ANI values were ~85% when *C. septicum* is compared with representative strains of the neighboring species, indicating clear species demarcation (Figure 2B). Finally, the *C. septicum* genomes were highly similar based on the DDH values that ranged from 96.7 to 97.9% for the five genomes in comparison to the *C. septicum* type strain DSM 7534^T^. On the other hand, the isDDH value of *C. septicum* DSM 7534^T^ compared to *C. chauvoei* genomes (DSM 7528^T^and JF4335) was 28.8. The GC content variability between the *C. septicum* genomes to the reference genome ranged from 0.09 to 0.35 whereas to *C. chauvoei* genomes, it was 0.43 and 0.44 (Supplementary Table S2). Taken together, these results indicate a clear species delimitation of *C. septicum* and confirm the stable phylogenetic relationship of *C. septicum* with *C. chauvoei*.

### CRISPR Regions

In strain DSM 7534^T^, we identified three CRISPR regions that were separated by a gene-encoding for IS256 family transposons. The CRISPR regions contained 28, 82, and 23 repeats, respectively, with the same repeat sequence (GTTTTATCTTAACTAGTGGAATGTAAAT). A CRISPR region containing 93 repeats was identified in the VAT 12 genome, while the draft genomes DRS014147 and RVDL_ALI carried 135 and 82 repeats, respectively. The CRISPR region of strain RVDL_ALI was split into two contigs. The genome P1044 harbored one CRISPR region with 79 repeats. Unique content of CRISPR spacer sequences was found in the all five genomes as depicted in Supplementary Figure S2. The repeat sequence (GTTTTATCTTAACTAGTGGAATGTAAAT) was present in all genomes.

Classification analysis of CRISPR-Cas systems revealed subtype I-B (Tneap–Hmari or CASS7) in all genomes, with Cas genes arranged as follow: *cas2*-*cas1b*-*cas4*-*cas3*-*cas5b*-*cas7i*-*cas8a1*-*cas6* (Makarova et al., 2011).

### Prophage Elements

**Figure 3:**
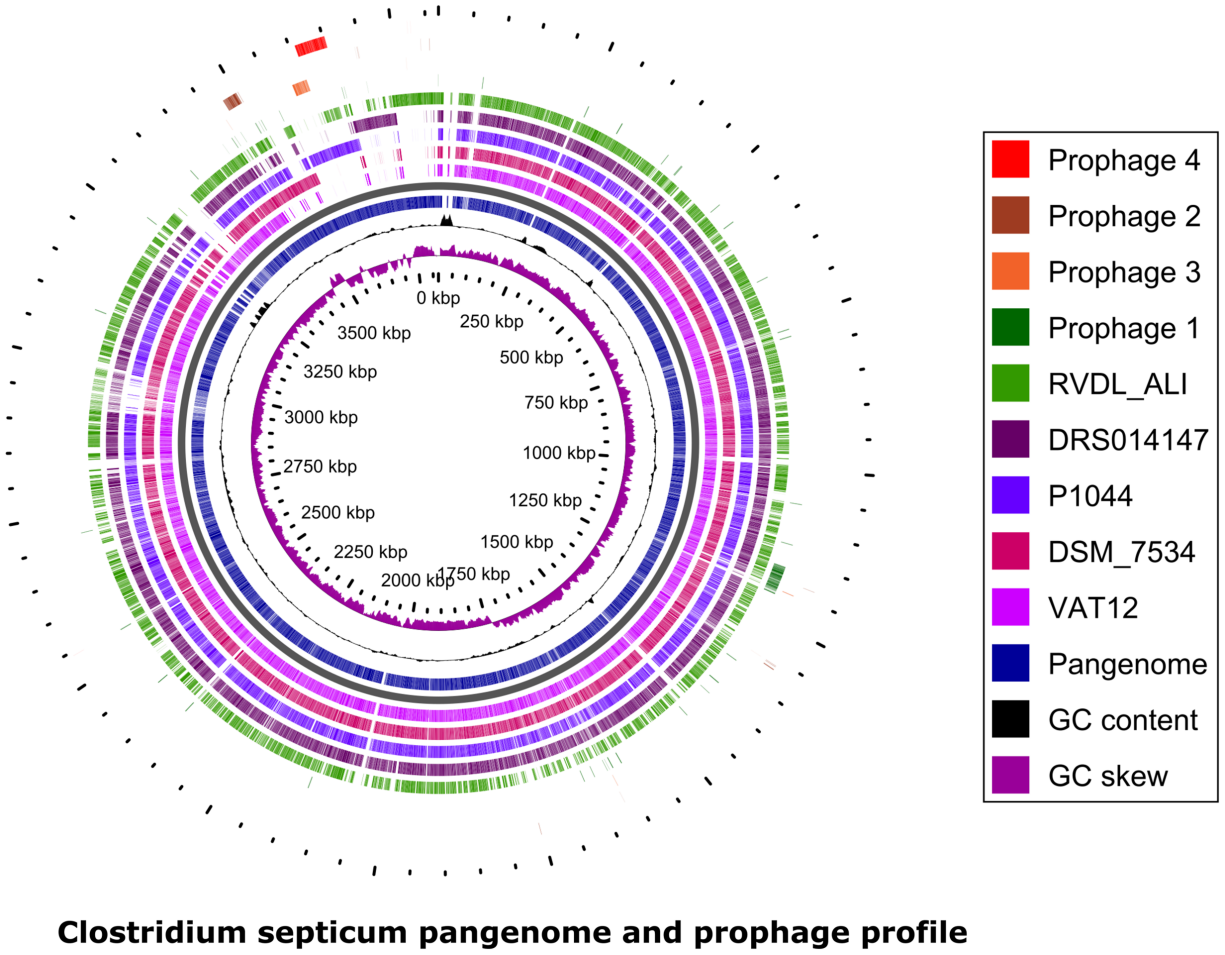
*Clostridium septicum* pangenome prophage profile. The pangenome plot depicts BLASTP relatedness among protein coding genes in five *C. septicum* genomes to species pangenome. The plot also represents the GC content and GCskew. Prophages (1–4) mapped to *C. septicum* pangenome shows the shared and unique presence of few phages in all strains or some strains, respectively. Pangenome prophage profile was created using GView Server.

Four different prophages (numbered 1 to 4) were predicted in the five genomes (Table 3, details Supplementary Table S3). The predicted prophages were less related to any reported prophages as indicted by the lower number and percentage values to first most common phage; 10.34% for Prophage 1, 16.66% for Prophage 2, 23.3% for Prophage 3, and 8% for Prophage 4. All prophages belonged to family Siphoviridae. Prophage 1 (47.7-kb) related to phiCD38-2/phiCD111/phiCD146 was present in all strains. Prophage vB_CpeS-CP51 (Prophage 2; 33.7-kb) was absent in VAT12. Prophage 3 (29.3-kb) related to phiCD6356 was present in the genome P1044 whereas prophage 4 (49.6-kb) related to Geobac_E2/Bacill_phIS3501/Coryne_StAB was shared among the genomes P1044 and RVDL_ALI. Schematic representation of prophages to *C. septicum* pangenome is depicted in Figure 3.

**Table 3:** Predicted prophages and their distribution across *C. septicum* genomes.

Prophage	Most common phages, NCBI accessions and BLAST hit genes count numbers	Distribution
Prophage 1 (47.7 Kb; CDS-58)	Clostr_phiCD38_2; NC_015568; 6 Clostr_phiCD111; NC_028905;6 Clostr_phiCD146; NC_028958; 6	All genomes
Prophage 2 (33.7 Kb; CDS-42)	Clostr_vB_CpeS_CP5; NC_021325; 7	All genomes except VAT12 and partially present in DRS01147
Prophage 3(29.3 Kb; CDS-30)	Clostr_phiCD6356; NC_015262; 7	P1044
Prophage 4 (49.6. Kb; CDS-45)	Geobac_E2; NC_009552; 4 Bacill_phIS3501; NC_019502; 4 Coryne_StAB; NC_048780; 4	P1044 and RVDL_ALI

*The most common phage homolog predicted is reported with the corresponding accession numbers and gene count*.

### Restriction-Modification (RM) Systems and Methylation Motifs

The complete genomes DSM 7534^T^ and VAT 12 share putative type I, II, and IV RM systems whereas a putative type III system was additionally present in DSM 7534^T^ strain. The type IV RM system encodes one restriction endonuclease (REase) as reported for the type IV RM systems for harboring only REases (Rusinov et al., 2015). The confirmed DNA methylations motifs of the identified RM systems were AA^m^GNNNNNRT^m^GAA and GT^m^ATA^m^C for both genomes and AGA^m^GC for the *C. septicum* DSM 7534^T^ genome (“A^m^” is the methylated base m6A; Table 4). The methylation motif summary analysis was carried out for the strain DSM 7534^T^ (Table 5). The total number of modifications identified for the *C. septicum* DSM 7534^T^ genome was 1,014,579 which included 66,811 4-methyl-cytosine (m4C), 14,780 6-methyl-adenine (m6A) and remaining were unidentified modifications types.

**Table 4:** Restriction-modification (RM) systems identified in *C. septicum* genomes RM systems involved, associated rrestriction endonucleases (REases) and methyltransferases (MTases), type/subtype information and recognition sequence they methylate, respectively, for DSM 7534^T^ and VAT 12 are indicated.

Putative *Clostridium septicum* RM systems				
Type	Gene	Name	Predicted recognition sequence	Coordinates
**DSM 7534; GenBank: CP023671 (3,399,422 bp)**
I	R	Csp7534IP	AAGNNNNNRTGAA	1,038,572–1,041,919 c
I	S	S.Csp7534I	AAGNNNNNRTGAA	1,042,179–1,043,366 c
I	M	M.Csp7534I	AAGNNNNNRTGAA	1,043,370–1,044,833 c
II	M	M.Csp7534ORF3775P		855,788–856,579
II	M	M.Csp7534III	GTATAC	1,644,132–1,645,619 c
II	R	Csp7534ORF7365P		1,681,333–1,682,523
II	M	M.Csp7534ORF7365P		1,683,072–1,684,316 c
II	M	M.Csp7534ORF13355P		2,904,200–2,904,955
III	R	Csp7534IIP	AGAGC	1,054,629–1,057,328 c
III	M	M.Csp7534II	AGAGC	1,057,344–1,059,206 c
IV	R	Csp7534ORF3610P		809,971–812,865 c
**VAT 12; GenBank: CP034358 (3,454,144 bp)**				
I	R	CseVAT12ORF15145P	AAGNNNNNRTGAA	3,229,337–3,232,441 c
I	S	S.CseVAT12ORF15145P	AAGNNNNNRTGAA	3,233,091–3,234,278 c
I	M	M.CseVAT12ORF15145P	AAGNNNNNRTGAA	3,234,282–3,235,745 c
II	M	M.CseVAT12ORF1960P	GTATAC	429,061–430,548 c
II	M	M.CseVAT12ORF8245P		1,710,236–1,710,991
II	M	M1.CseVAT12ORF9305P		1,939,531–1,940,778
II	M	M2.CseVAT12ORF9305P		1,940,771–1,941,658
IV	R	CseVAT12ORF14140P		2,996,256–2,999,150 c

**Table 5:** Methylated motifs detected in *C. septicum* DSM 7534^T^ genome.

Motif	Modified position	Type of Modification	Methylated motifs detected (%)	Number of motifs detected	Number of motifs in the genome	Mean modification QV^a^	Mean motif coverage^b^	Inverse complementary motif
AGAGC	3	m6A	99.92	3,808	3,811	177.1	114.82	-
TTCAYNNNNNCTT	4	m6A	95.62	720	753	138.28	116.27	AAGNNNNNRTGAA
AAGNNNNNRTGAA	2	m6A	94.02	708	753	137.64	108.02	TTCAYNNNNNCTT
GTATAC	5	m6A	95.31	549	576	137.97	115.61	GTATAC
G	1	Unknown	34.49	326,979	948,116	50.96	113.76	-
CSVV	1	m4C	25.55	21,010	82,242	50.06	125.94	-
ABDYAGYA	1	m6A	18.4	1,112	6,042	47.23	116.87	-
TVVVDYNH	1	Unknown	8.63	18,721	217,022	38.26	123.5	-

*Motif recognized, position, type of modifications (m6A, m4C and unknown), methylated motifs (%) among all motifs detected for the DSM 7534 T are depicted*.

a *Mean Modification QV refers to the level of confidence that a base is methylated. A QV of 30 or higher is considered significant*.

b *Mean coverage for all instances where this motif was detected as modified*.

### Comparative Genomics and Phylogeny

**Figure 4:**
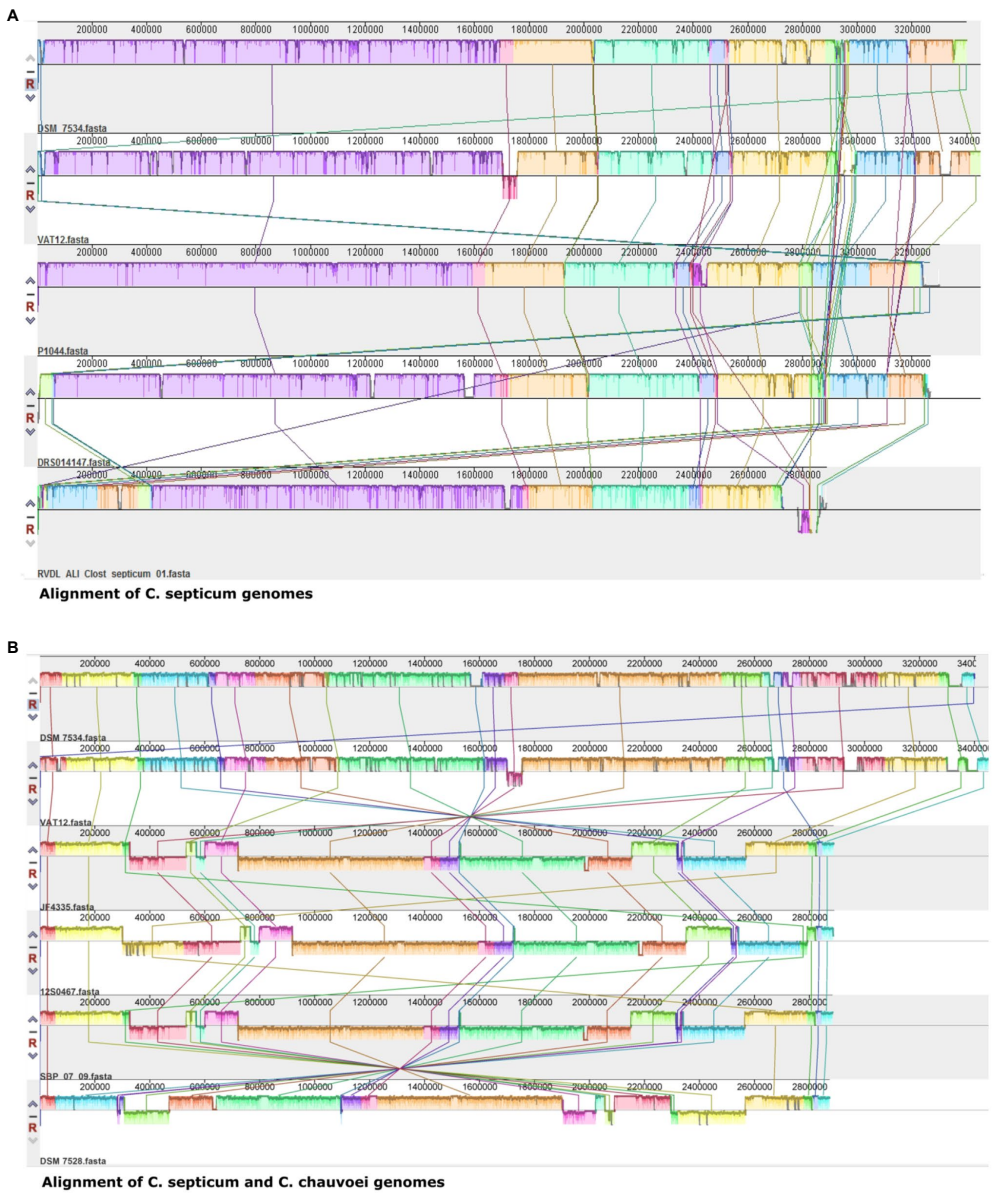
Multiple genome alignment with progressiveMauve shows the presence of locally collinear blocks (LCBs) among the genomes. **(A)** Alignment of all *C. septium* genomes. **(B)** Alignment of complete *C. septium* (DSM 7534^T^ and VAT 12) and *C. chauvoei* (DSM 7528^T^, JF4335, 12S0467, and SBP 07/09) genomes. The sharing of LCBs indicates regions of conservation among genomes.

Genome alignment of the five *C. septicum* genomes revealed 15–17 conserved local collinear blocks (LCBs; Figure 4A). In addition, genome alignment of *C. septicum* and *C. chauvoei* revealed conserved LCBs (Figure 4B). These LCBs indicate regions of conservation among genomes and unshared regions indicate unique genetic elements.

Orthologous gene clustering with Panaroo (Tonkin-Hill et al., 2020) predicted 3,740 genes, comprising the pangenome of the five genomes. Of these, 2,311 (core genome) were present in all genome datasets while 1,429 represented the accessory genome. The COG annotation indicated that most of the accessory genes belonged to the COG category representing function unknown (S; 215 genes) followed by replication, recombination and repair (L; 182 genes; Supplementary Figure S3). Some of the accessory genetic elements were also contributed by prophages (Figure 3). Of the core genome, 1,400 (61.1%) genes were assigned to a specific functional category in the KEGG database, of which 371 were associated with metabolic pathways. Further 197 proteins represent the processing of genetic information, followed by 172 proteins for carbohydrate metabolism. The list of 32 metabolic pathway modules (complete) representing different metabolic processes identified in the *C. septicum* core genome is shown in Supplementary Table S4. Of the accessory genes, only 317 genes (24.5%) were annotated using the KEGG database, including 61 proteins for signaling and cellular processes, 52 for genetic information processing, and 33 for environmental information processing. Fifty-three accessory genes were predicted to encode for metabolic pathways. Only one complete pathway module for central carbohydrate metabolism was predicted for accessory genome.

**Figure 5:**
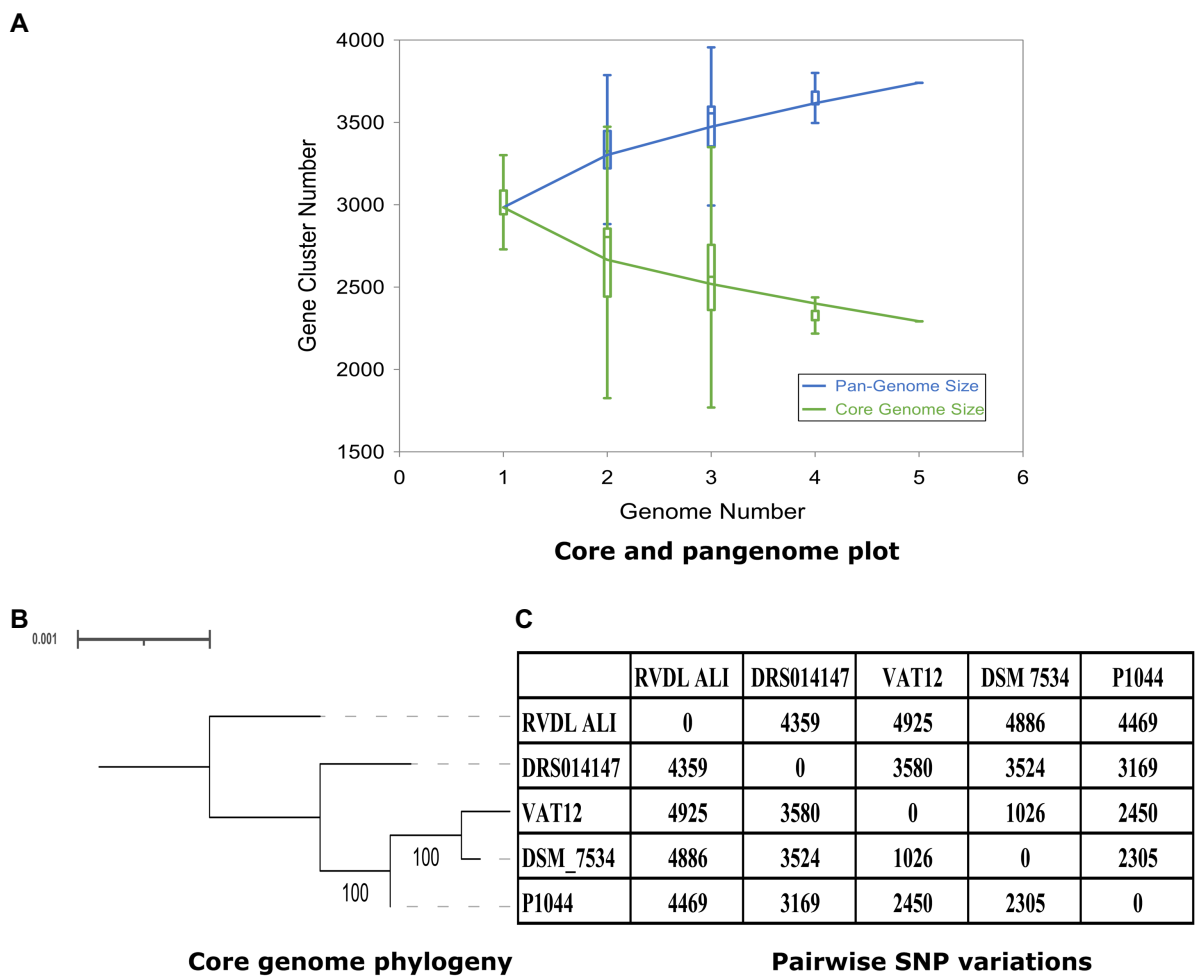
Pangenome, core genome plots and core genome phylogeny. **(A)** The pan-genome and core genome plot. Pangenome analysis indicated 2,311 and 1,429 core and accessory genes, respectively, and the pangenome profile indicated and open pangenome for *C. septicum*. **(B)** Core genome phylogeny based on 2,311 genes. **(C)** Pairwise SNP variations with in the core genome.

The pan-genome trajectory pattern showed a large expansion of the pangenome and a decrease in the core genomes. The fit for the pangenome profile curve using the power-law regression model resulted in *B* = 0.01 (*r*^2^ = 0.999), confirming the openness of the pangenome (Figure 5A). Core genome phylogeny based on 2,311 genes (2,234,611-bp) revealed 7,870 core variable positions in the alignment with 2,976 of them were parsimony informative. The genomes of strains DSM 7534^T^ and VAT12 were also phylogenetically related with 100% bootstrap values, while the genomes of strains RVDL_ALI and DRS014147 were observed as singletons (Figure 5B). Pairwise SNP variations between the genomes ranged from 1,026 (DSM 7534^T^ and VAT12) to 4,886 SNPs (DSM 7534^T^ and RVDL_ALI; Figure 5C).

### Virulence Genes

#### *Clostridium septicum* α-Toxin

The *C. septicum* α-toxin gene (*csa*) was present in all genomes and showed pairwise sequence identity of over 99.9%, with only a single synonymous SNP (T/C) observed at position 279. The genome RVDL_ALI had however four additional SNPs, two of which were nonsynonymous (Supplementary Figure S5A). To further investigate the sequence conservation of the *csa*-gene on a larger data set, we downloaded 29 *csa* sequences with complete CDS from NCBI. Sequence comparison revealed a sequence identity of more than 99% except for three strains with 97 to 98%. Phylogenetic analysis of the *csa* genes showed clustering of strain RVDL_ALI with two strains also isolated from cattle (Alhassa1 and Yamaguchi 6335). Strains DSM 7534^T^ and VAT12 clustered with a strain from Japan (Tokachi). Strains DRS014147 and P1044 clustered with strains from Japan (Fukushima 5) and China (AY829447.1; Supplementary Figure S4).

#### *Clostridium septicum* Novel Toxin Homolog

**Figure 6:**
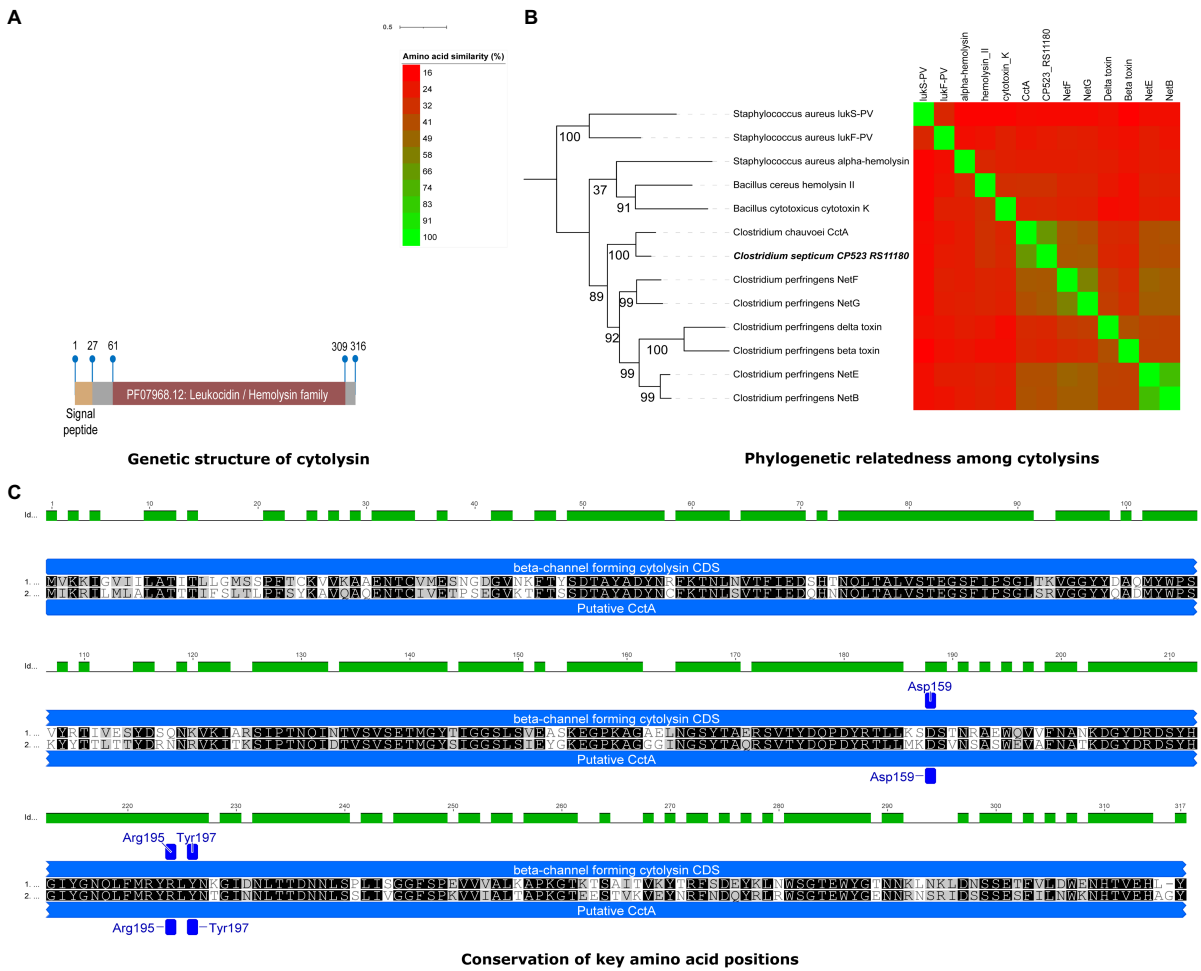
Novel cytolysin identified in *C. septicum* genomes. **(A)** Genetic structure of novel cytolysin identified in *C. septicum* with respect to genetic structure such as signal peptide and Leucocidin/Hemolysin toxin domain. **(B)** Phylogenetic relatedness of cytolysin to *C. chauvoei* CctA (100% boostrap support value) and other pore forming toxins in related species. **(C)** Conservation of key amino acid positions in *C. septicum* cytolysin (upper) with *C. chauvoei* CctA (lower) reported for virulence.

A gene encoding a novel toxin homolog was identified in all *C. septicum* genomes. The predicted protein contains a leukocidin/hemolysin domain similar to beta-channel forming cytolysin reported for other *Clostridium* species. In particular, it shares 71.10% aa identity with *C. chauvoei* toxin A (CctA; Table 6) that is a potent cytolysin involved in *C. chauvoei* virulence in blackleg disease in cattle and sheep (Frey and Falquet, 2015). The predicted protein also has the exact same size as CctA (317 aa). In addition, phylogenetic analysis with several pore-forming toxins revealed a closer relationship to CctA, with 100% bootstrap support, than to other *Clostridium* pore-forming toxins, including *C. perfringens* beta, NetB, NetE, NetG, NetF, and delta toxins (Figure 6B). We designated the identified homolog, *Clostridium septicum* toxin A (CstA). Sequence analysis of CstA-encoding gene (*cstA*) revealed 100% sequence identity in all *C. septicum* genomes.

**Table 6:** Virulence factors predicted for *Clostridium septicum* strains.

Product description	Locus tag (DSM 7534^T^)	CDS length	Gene homolog in *C. chauvoei*	Locus tag (DSM 7528)	CDS length	% Pairwise identity (CDS)
Alpha toxin	CP523_RS04890	440	-			-
Sialidase	CP523_RS01755	1,297	Nan A Sialidase	BTM21_RS07230	1,300	71.67%
Cytolysin	CP523_RS11180	317	*Clostridum chauvoei* toxin A (CctA)	BTM21_RS09230	312	71.10%
Hemolysin D	CP523_RS10485	222	Hemolysin D	BTM21_RS01165	223	79.37%
Hemolysin III	CP523_RS08010	216	Hemolysin III	BTM21_RS00225	216	91.204%.
Hemolysin A	CP523_RS15115	270	Hemolysin A	BTM21_RS05040	270	92.222%.
Hyaluronidase NagH	CP523_RS05150	1888	Hyaluronidase NagH	BTM21_RS10205	1887	86.18%
Hyaluronidase NagJ	CP523_RS04225	1,319	Hyaluronidase NagJ	BTM21_RS09515	1,321	83.39%
Collagenase	CP523_RS01750	983	Collagenase	BTM21_RS07225	968	77.52%

*The primary virulence factors predicted in Clostridum septicum strain DSM 7544 ^T^ showing significant homology to C. chauvoei DSM 7524^T^ strain are shown. The similarities in both species were calculated based on the percentage of pairwise BLASTP identity of CDS*.

The CstA protein sequences include a signal peptide region with 27 aa residues (Sec/SPI) followed by a leucocidin/hemolysin domain (aa position 69 to 309). The cleavage site A of signal peptidase I (Tat/SPI) was between nucleotide positions 27 and 28 (ATA-TT; Figure 6A). Three conserved aa residues (Asp159, Arg195, and Tyr197) reported by Frey et al. (2012) in the mature protein of *C. chauvoei* CctA were also identified in CstA. These aa residues are important for membrane specificity and activity of the pore-forming toxins, *C. perfringens* NetB and beta toxin, and *S. aureus* alpha toxins (Song et al., 1996; Steinthorsdottir et al., 1998; Nagahama et al., 1999). The conserved aa positions for the predicted CstA might therefore indicate similar pore-forming activity (Figure 6B).

ABCpred server predicted 17 and 19 linear epitopes for the CctA and CstA, respectively. Considering the score of predicted epitopes (indicative of epitope potency), CstA was predicted with more epitopes with the highest scores (three epitopes with 0.89 score) as compared to CctA (1 epitope with 0.89 score). Also, the cumulative total score when all epitopes above cutoff value 0.75 are considered was higher for CstA (total score 15.53) as compared to CctA (total score 13.92; Supplementary Table S5).

#### *Clostridium septicum* Sialidases

Genes encoding sialidases were identified in the *C. septicum* strains (Table 6). The *C. septicum* sialidase gene encodes 1,296 aa (EC 3.2.1.18), exhibiting 71.6% aa identity to NanA sialidase of *C. chauvoei*. The predicted protein sequence of sialidase was conserved; three aa variations in the genome DRS014147, one aa variation for RVDL_ALI. The latter had also a premature stop codon, resulting in a shorter predicted protein (1,237 aa; Supplementary Figure S5B).

#### *Clostridium septicum* Hemolysin

Three genes (locus tags CP523_RS10485, CP523_RS08010, and CP523_RS15115) encoding hemolysin proteins were identified in the *C. septicum* genomes (Table 6). CP523_RS10485 was similar to the *C. chauvoei* hemolysin D (79.3% aa identity; Frey and Falquet, 2015), CP523_RS08010 was similar to *C. chauvoei* hemolysin III (91.2% aa identity), and CP523_RS15115 was similar to *C. chauvoei* hemolysin A (FtsJ RNA methyltransferase; 92.2% aa identity; Frey and Falquet, 2015). The *C. septicum* hemolysin genes were detected in all five genomes with 100% sequence identity, except for the hemolysin A gene in the RVDL_ALI genome, which showed one aa variation (Supplementary Figure S5C).

In addition, a gene (CP523_RS13325) with very limited homology (16% at aa level) to *C. chauvoei* hemolysin *XhlA* gene (Frey and Falquet, 2015) was identified in four *C. septicum* genomes showing 100% identity at aa level.

#### *Clostridium septicum* Hyaluronidases

*Clostridium septicum* genomes harbored homologs for hyaluronidases NagH and NagJ reported in *C. chauvoei* (Frey and Falquet, 2015). The hyaluronidases genes were identified in the five *C. septicum* genomes with a pairwise aa sequence identity of 99.6 and 99.7% for NagH and NagJ, respectively Supplementary Figures S5D,E.

#### *Clostridium septicum* Collagenase

A gene coding for collagenase (EC 3.4.24.3) was predicted in *C. septicum* genomes with identical aa sequences except for RVDL_ALI genome which had a short collagenase gene.

#### *Clostridium septicum* Flagellin

The genome sequence of *C. septicum* DSM 7534^T^ and P1044 revealed four complete flagellin (*fliC*) genes, and a similar gene presence with reduced homology was also seen in the other strains. The four *fliC* genes showed a considerable homology of 95.4% pairwise identity at aa level and had a central variable region.

## Discussion

Clostridial gas gangrene (myonecrosis) is a lethal infection that affects a wide range of hosts with the involvement of different pathogenic species, which may influence the symptoms and clinical course of the disease. Potent exotoxins released by these pathogens in the host tissues are thought to be a major contributor to the observed pathogenesis (Stevens et al., 2012; Carter et al., 2014; Oda et al., 2015; Yamamura et al., 2019). *Clostridium perfringens* and *C. septicum* are the most commonly isolated organisms in these infections. However, unlike *C. perfringens*, detailed genomic characterization of *C. septicum* has not been yet performed. The study reports the first complete sequence of the *C. septicum* type strain (DSM 7534^T^). In addition, a comparative analysis involving all available *C. septicum* genomes (*n* = 5) was performed in order to decipher genomic and virulence aspects that are possibly associated with the development of gas gangrene in humans and animals. The size of the circularized *C. septicum* genomes (3.3–3.4-Mb) was similar to *C. perfringens* (3–3.5-Mb) but slightly larger than the phylogenetically related species, *C. chauvoei* (2.8-Mb; Shimizu et al., 2002; Falquet et al., 2013; Rychener et al., 2017; Table 2). The DSM 7534^T^ genome also harbored a small plasmid (5.2-kb) similar to that reported for *C. chauvoei* (4.1-kb; Falquet et al., 2013; Thomas et al., 2017). The plasmid also encoded a viroplasmin family protein and Teicoplanin resistance protein (vanZ; Figure 1). Previous studies have reported RNase H1/viroplasmin domain-containing protein associated with Caulimovirus (Volovitch et al., 1990), whereas the role of the similar protein in bacteria is not reported so far. The vanZ protein is associated with teicoplanin resistance and the gene orthologs have been reported from several bacterial genera (Vimberg et al., 2020). A previous study has shown clinical resistance to vancomycin in two strains recovered from clinical cases (Aldape et al., 2018). The vancomycin resistance cassette was also reported to be encoded on a 5-kb plasmid (Aldape et al., 2018). The present study also shows the existence of vanZ protein gene on a 5-kb plasmid similar to the report.

Genome sequencing provides a better resolution for depicting phylogenetic and taxonomic relatedness of bacterial genomes compared to 16S rRNA sequence analysis. The taxonomical classification of *C. septicum* was congruent between genome-based approaches and previous reports using 16S rRNA sequences (Kuhnert et al., 1996). The results indicate a clear species delimitation of *C. septicum* and confirm its phylogenetic relationship with *C. chauvoei*, as previously reported (Kuhnert et al., 1996).

*C. septicum* had CRISPR elements present in all genomes. This was similar to *C. chauvoei*, but different to *C. perfringens*, where studies showed the absence of CRISPR regions in many strains (Long et al., 2019). The CRISPR repeat arrays were interrupted by two mobile elements in strain DSM 7534T, which was also found in *C. chavuoei* (Rychener et al., 2017). Interestingly, the *C. septicum* genomes also showed genetic variability for the CRISPR array and spacers. This heterogeneity of CRISPR spacer sequences may provide a basis for genotyping *C. septicum* strains, as previously shown for related pathogen such as *C. chauvoei* (Rychener et al., 2017) and *C. difficile* (Andersen et al., 2016). Indeed, the diversity of *C. septicum* CRISPR spacer arrays showed a good correlation with the core genome phylogeny (Supplementary Figure S2). Hence, a CRISPR spacer-based typing approach may be another option for strain typing of this species. However, to achieve this, sequence data will need to be generated from more strains obtained under different geographic, host, and disease conditions. These will provide further insight into genetic diversity and could facilitate the identification of specific host and disease associations in *C. septicum*.

*C. septicum* genomes were predicted to encode four prophages (Table 3). Prophage 1 and 3 matched to phiCD38-2/phiCD111/phiCD146 and phiCD6356 phages respectively, reported earlier from *C. difficle* (Fortier and Moineau, 2007; Sekulovic et al., 2014). On the other hand, prophages (Geobac_E2/Bacill_phIS3501/Coryne_StAB) identical to prophage 4 are also reported in the genus *Clostridium* (Smith et al., 2021) as well as in other species such as *Shigella* (Muthuirulandi Sethuvel et al., 2019) and *Corynebacterium* (NC_048780). Phage vB_CpeS-CP51 (prophage 2), has been initially recognized as a temperate bacteriophage of *C. perfringens* (Gervasi et al., 2013) and was later reported also in *C. chauvoei* (Frey and Falquet, 2015). The presence of relatively higher numbers of prophages compared to *C. chauvoei* (Thomas et al., 2017), as well as the presence of CRISPR elements in all *C. septicum* genomes analyzed, likely indicates limited functionality of CRISPR elements in phage inhibition. This is however similar to *C. perfringens*, in which no direct correlation was found between the prophage frequencies and the absence or presence of CRISPR elements (Abdel-Glil et al., 2021).

RM systems include a restriction endonuclease (REase) and a modification methyltransferase (MTase). The REase degrades DNA from any exogenous source, whereas the MTase methylates the host REase target sites in the genome and thus protects them from cleavage (Blow et al., 2016). The RM system detected for the two circularized genomes showed variations with respect to the RM system types they carried (Table 4). Highly variable RM systems were identified in 302 environmental and outbreak-associated *Listeria monocytogenes* strains, with type II followed by type I and other types (III and IV) predominating, indicating a wide diversity of RM systems even within the same species (Chen et al., 2017). The RM system of *C. chauvoei* was conserved among three strains and included type I, II, and IV systems (http://rebase.neb.com/rebase/rebase.html; assessed April 14, 2021). In contrast, RM systems in *C. perfringens* strains (*n* = 62 strains) were diverse, ranging from zero to all combinations of RM systems (Type I to IV systems).^7^ The characterized *C. septicum* strain (DSM 7534^T^) harbored methyltransferase for m6A methylation. In addition, an absence of m5C methylation was observed in *C. septicum* (Table 5), reflecting either the true absence or the lower sensitivity of SMRT sequencing to detect m5C methylation (Murray et al., 2012).

Pangenome analysis based on orthologous gene clustering for the five genomes revealed an open pangenome, with the accessory genome comprising 1,429 genes (Figure 5). Most of the accessory genes belonged to the replication, recombination and repair category or to the unknown function category in COG annotation (Supplementary Figure S3). This suggests a role of horizontal genetic transfers in shaping the composition of the accessory genome of *C. septicum*. Similar results were reported for *C. perfringens*, wherein 849 genes within the accessory genome in contrast to 79 genes within the core genome belonged to replication, recombination, and repair mechanism (Kiu et al., 2017). A comparative genomic study of 206 genomes of *C. perfringens* strains showed that the major phylogroups exhibit a collinear distribution pattern of accessory genes, indicating the possible relevance of specific accessory genes in bacterial specialization (Abdel-Glil et al., 2021). The accessory genetic elements in *C. septicum* are resembling in frequency and COG category those in the genomes of *C. perfringens*. This contrasts with the limited accessory genetic variability found in *C. chauvoei*, the species most closely phylogenetically related to *C. septicum* (Rychener et al., 2017; Thomas et al., 2017). All core metabolic pathways with respect to energy, carbohydrate, amino acids, nucleotides and cofactor, and vitamins were represented in the core genome (Supplementary Table S4). Similar presence of high number of genes involved in carbohydrate metabolism was represented in the core genome of *Clostridium butyricum* (Zou et al., 2021).

The *Clostridium septicum* alpha toxin (*csa*) gene was mostly conserved in the analyzed five genomes, but variations were observable with other published *csa* genes (Supplementary Figure S4). A previous study reported seven unique patterns in the deduced α-toxin aa sequences of 25 *C. septicum* strains. The same study also reported unique insertion, altered stop codon position, and deletion of 9-bp for the involved strains (Amimoto et al., 2006). In the present study, similarly, a higher genetic variation was found between strains for α-toxin.

Besides the α-toxin, a novel putative cytolysin gene, *Clostridium septicum* toxin A (CstA) present in all *C. septicum* genomes was identified. It showed 71.1% aa identity to the CctA gene of *C. chauvoei*, predicted to encode a similar signal peptide and Leucocidin/Hemolysin toxin domains (Figure 6). Strong conservation of the CctA gene between *C. chauvoei* strains has been reported (Rychener et al., 2017), and the genomes of the current study also showed similar conservation (100%) for the putative cytolysin gene in *C. septicum*. Previous studies have demonstrated CctA as one of the main protective antigens in vaccines against blackleg (Frey et al., 2012), and a recent study reported the production of neutralizing antibodies against CctA following the blackleg vaccination (Nicholson et al., 2019). The presence of analogous potent B-cell epitopes predicted for the *C. septicum* CstA (Supplementary Table S5) suggests that it is suitable as another potent vaccine target candidate for clostridial infections, in addition to known toxins such as α-toxin (Haghroosta et al., 2020). It must be noted that a toxin known as septicolysin (delta-toxin) belong to cholesterol-dependent cytolysin (CDC) family has been proposed to have a synergistic effect with α-toxin in the pathogenesis of *C. septicum* infection (Popoff, 2016). Septicolysin associated hemolytic activity was reported to be less than 5% of the total detectable hemolytic activity and the remaining was attributed to α-toxin in *C. septicum* (Ballard et al., 1992). Based on a published *Clostridium septicum* partial septicolysin (*spl*) gene sequence (AJ539084.1), we did a BLASTN query with all five *C. septicum* CDSs. There were no homologous counterparts identified within the strains with high identity. This *spl* gene had 78.7% nucleotide identity with another gene encoding for an amino acid permease present in all strains (Locus tag: CP523_RS09335 for DSM 7534^T^). Interestingly, the *spl* gene sequence is 53.1% similar to the identified CstA indicating that this novel cytolysin homolog (CstA) is divergent from the *C. septicum* streptolysin (delta toxin) previously reported.

Between the species *C. chauvoei* and *C. septicum*, both cytolysin and sialidase homologs showed reduced similarity (<75%), whereas the hyaluronidase genes (NagH and NagJ), hemolysins and collagenase showed higher similarity (Table 6). This suggests that speciation likely had a smaller effect on genetic divergence of other virulence genes than for cytolysin and sialidase genes. Among the virulence factors identified in the genomes of both species, the predominant classes were sialdiases, hyaluronidases, hemolysins, leucocidin, and aerolysin family toxins (CctA in the case of *C. chauvoei* and CstA and α-toxin in the case of *C. septicum*), as well as collagenase and internalin. With the known and predicted virulence factors, *C. septicum* was unique to harbor the α-toxin gene as compared to *C. chauvoei*. In summary, our analysis revealed a core set of putative virulence-related genes likely involved in *C. septicum* disease progression, as most of the identified genes encode enzymes that act extracellularly, and some of these homologs, particularly CctA, have been significantly associated with blackleg pathogenesis in cattle.

## Conclusion

To conclude, this is the first comparative genomic study for the *C. septicum* species using five genomes. Although our analysis included all publicly-available genomes of *C. septicum* in NCBI, the small number of available genomes may have made it difficult to portrait the overall population diversity of the species, which may be considered a limitation of our analysis. Nevertheless, the analysis described here represents a starting point for understanding the genomic variation of *C. septicum* that is a relevant human and animal pathogen. The high fatality rate of diseases caused by this bacterium will motivate further studies to sequence its genome and more deeply investigate its genetic properties. The analysis presented here will be useful for future studies in several ways. First, the taxonomic classifications based on 16S rRNA and genome-wide approaches reconfirm the phylogenetic proximity to *C. chauvoei*. Second, unlike *C. chauvoei*, a less variable species, the genomes of *C. septicum* strains exhibited high genetic diversity in terms of prophages, CRISPR spacers, RM systems, accessory genomes, and core genome SNPs, possibly due to the diverse lifestyle and broad host range. Third, *C. septicum* encodes several virulence factors that are likely to be involved in the clinical course of *C. septicum* disease. These virulence factors were genetically most closely related to those of *C. chauvoei*. Most importantly, a novel toxin homolog CstA was identified that resembled the *C. chauvoei* key virulence factor CctA. The role of the virulence factors predicted in this study needs further investigation in *in vitro* and *in vivo* models to understand the disease and pathogenesis occurring during *C. septicum* infections in animals and humans.

## Data Availability Statement

The original contributions presented in the study are publicly available. The sequence data generated for this study can be found in the National Center for Biotechnology Information Reference Sequences (RefSeq) using accession numbers, NZ_CP023671.1 (chromosome) and NZ_CP023672.1 (plasmid; https://www.ncbi.nlm.nih.gov/bioproject/412368).

## Author Contributions

PT and MA-G conceptualized and designed the study, performed the bioinformatic analysis, and wrote the manuscript. AS and AB provided support for the bioinformatic analysis. IE performed the next-generation sequencing. LW, HN, and MP supervised the study. CS conceptualized and supervised the study and critically revised and improved the manuscript. All authors contributed to the article and approved the submitted version.

## Funding

AB was supported by the Deutsche Forschungsgemeinschaft (German Research Foundation) under Germany’s Excellence Strategy (EXC 2051, project 390713860).

## Conflict of Interest

The authors declare that the research was conducted in the absence of any commercial or financial relationships that could be construed as a potential conflict of interest.

## Publisher’s Note

All claims expressed in this article are solely those of the authors and do not necessarily represent those of their affiliated organizations, or those of the publisher, the editors and the reviewers. Any product that may be evaluated in this article, or claim that may be made by its manufacturer, is not guaranteed or endorsed by the publisher.

## Footnotes

^1^ https://github.com/tseemann/shovill

^2^ https://github.com/tseemann/barrnap

^3^ http://rebase.neb.com

^4^ https://server.gview.ca/

^5^ https://github.com/tseemann/abricate

^6^ https://github.com/tseemann/snp-dists

^7^http://rebase.neb.com/rebase/rebase.html (Assessed April 14, 2021).

## Supplementary Material

Supplementary Figure S1 Taxonomic classification of *C. septicum* based on 16srRNA gene. The 16S rRNA gene based phylogenetic analysis of *C. septicum* indicates close relatedness with *C. chauvoei* within *Clostridium* genus cluster 1 (*Clostridium sensu stricto*).

Supplementary Figure S2 Diversity and structure of CRISPR regions and CRISPR spacers. CRISPR spacers present among strains are represented in the form of an array. (A) Every unique spacer present within any strain is represented with different color codes. (B). Spacers that are shared among strains are represented with same color codes and unshared spacers are represented in gray. The three CRISPR regions in the type strain DSM 7534^T^ intervened by IS256 family transposons are indicated as white space.

Supplementary Figure S3 Clusters of Orthologous Groups (COGs) categorization of core and accessory genes. Functional annotation of the core and accessory genes were carried out for COGs. Most of the accessory were belonging to the category of representing function unknown (S) and replication, recombination and repair (L).

Supplementary Figure S4 Phylogenetic tree involving *C. septicum* alpha toxin cds at nucleotide level. Phylogenetic relatedness of alpha toxin gene (full CDS) among *C. speticum* strains from diverse host/source and geographical sources was inferred.

Supplementary Figure S5 Protein alignment of *Clostridium septicum* virulence factors. Alignment of primary virulence factors showing amino acid variations are shown in Figure S5. (A) Alpha toxin, (B) Sialidase, (C) Hemolysin A, (D), Hyaluronidase NagJ and (E) Hyaluronidase NagH.

Supplementary Table S1 Metadata summary of sequences retrieved from NCBI. Details with respect to species, strain name, and NCBI accession numbers of species of Clostridium sensu stricto used for 16S rRNA phylogenetic analysis are shown.

Supplementary Table S2 Taxonomic classification of *C. septicum* based on in silico DNA-DNA hybridization.

Supplementary Table S3 Prophages identified in *C. septicum* genomes. The prophages identified in five *C. septicum* strains indicating the size in bases, score predictions based on PHASTER, protein coding genes and BLAST identity are given.

Supplementary Table S4 KEGG pathway modules identified in the core genome of *C. septicum*.

Supplementary Table S5 Predicted B-cell epitopes in *C. chauvoei* toxin A (CctA) and in the identified homolog, *C. septicum* toxin A (CstA) identified by ABCpred server.
