# Supplementary figures and images for: First Comparative Analysis of Clostridium septicum Genomes Provides Insights Into the Taxonomy, Species Genetic Diversity, and Virulence Related to Gas Gangrene
Source: Front Microbiol. 2021 Dec 9;12:771945. doi: 10.3389/fmicb.2021.771945 (PMC8696124; doi:10.3389/fmicb.2021.771945)

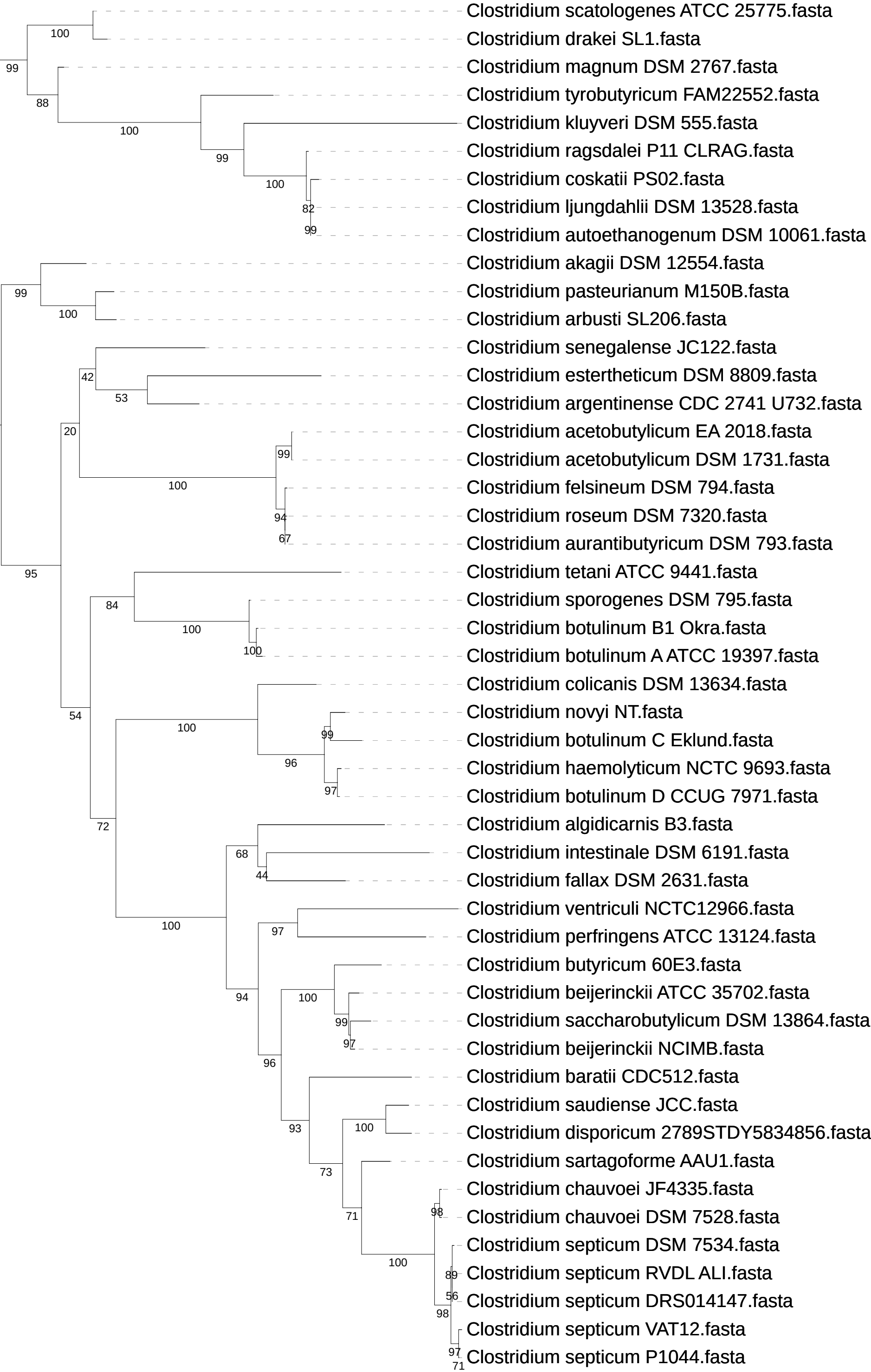

Supplement: Supplementary Figure S1 — Taxonomic classification of C. septicum based on 16srRNA gene. The 16S rRNA gene based phylogenetic analysis of C. septicum indicates close relatedness with C. chauvoei within Clostridium genus cluster 1 (Clostridium sensu stricto). [file Data_Sheet_1.PDF]

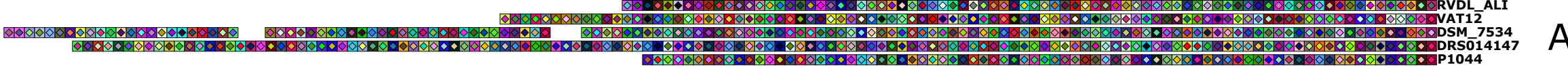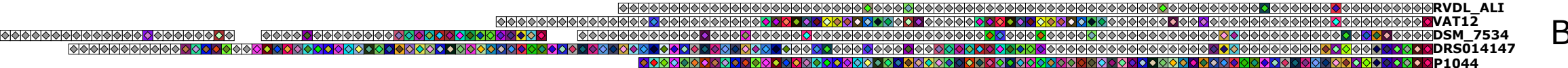

Supplement: Supplementary Figure S2 — Diversity and structure of CRISPR regions and CRISPR spacers. CRISPR spacers present among strains are represented in the form of an array. (A) Every unique spacer present within any strain is represented with different color codes. (B). Spacers that are shared among strains are represented with same color codes and unshared spacers are represented in gray. The three CRISPR regions in the type strain DSM 7534T intervened by IS256 family transposons are indicated as white space. [file Data_Sheet_2.PDF]

## COG Classifications

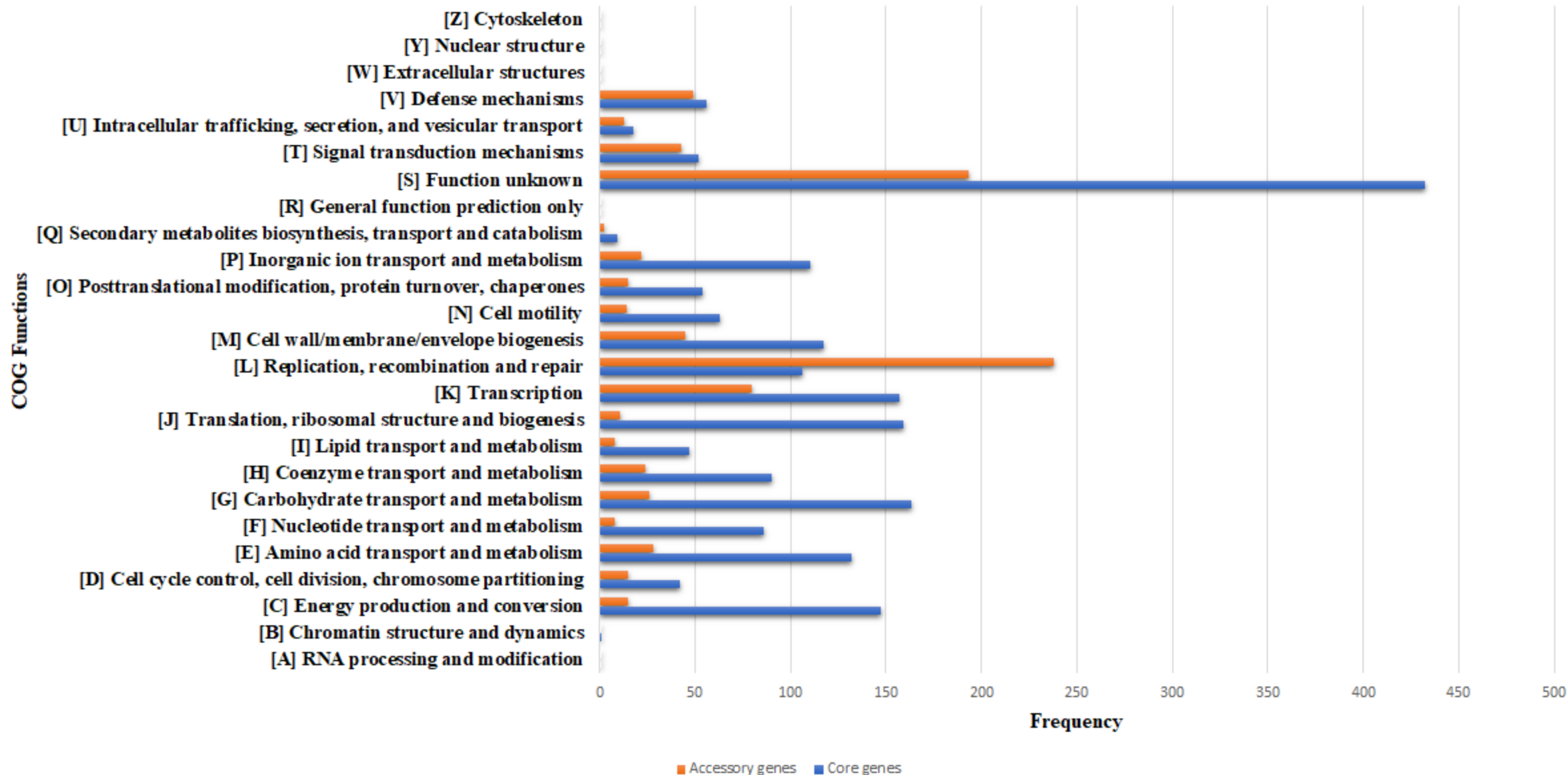

Supplement: Supplementary Figure S3 — Clusters of Orthologous Groups (COGs) categorization of core and accessory genes. Functional annotation of the core and accessory genes were carried out for COGs. Most of the accessory were belonging to the category of representing function unknown (S) and replication, recombination and repair (L). [file Data_Sheet_3.PDF]

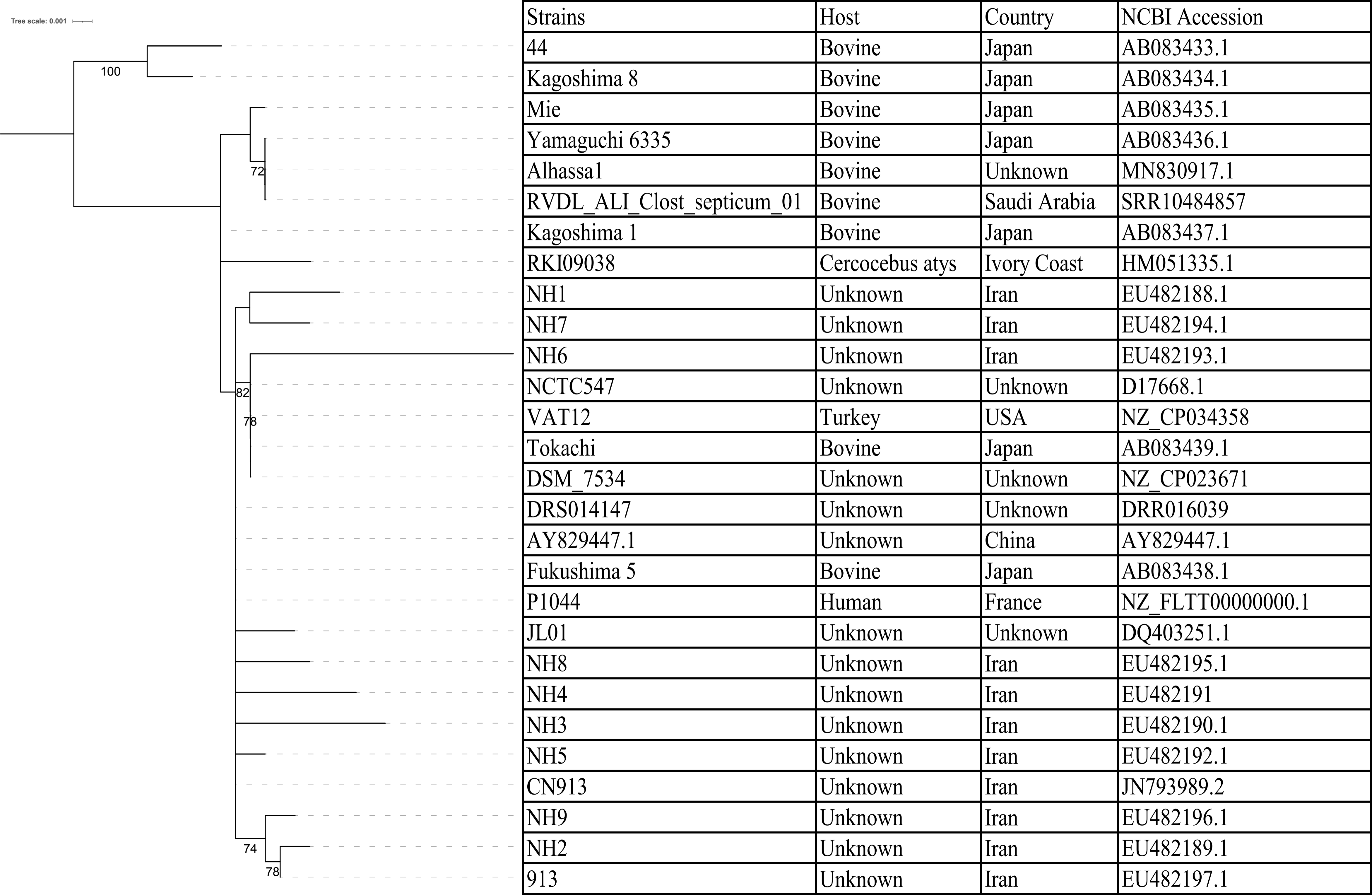

Supplement: Supplementary Figure S4 — Phylogenetic tree involving C. septicum alpha toxin cds at nucleotide level. Phylogenetic relatedness of alpha toxin gene (full CDS) among C. speticum strains from diverse host/source and geographical sources was inferred. [file Data_Sheet_4.PDF]
