# Supplementary material for: First Comparative Analysis of Clostridium septicum Genomes Provides Insights Into the Taxonomy, Species Genetic Diversity, and Virulence Related to Gas Gangrene
Source: Front Microbiol. 2021 Dec 9;12:771945. doi: 10.3389/fmicb.2021.771945 (PMC8696124; doi:10.3389/fmicb.2021.771945)
Supplement: Supplementary Figure S5 — Protein alignment of Clostridium septicum virulence factors. Alignment of primary virulence factors showing amino acid variations are shown in Figure S5. (A) Alpha toxin, (B) Sialidase, (C) Hemolysin A, (D), Hyaluronidase NagJ and (E) Hyaluronidase NagH. [file Data_Sheet_5.PDF]

A

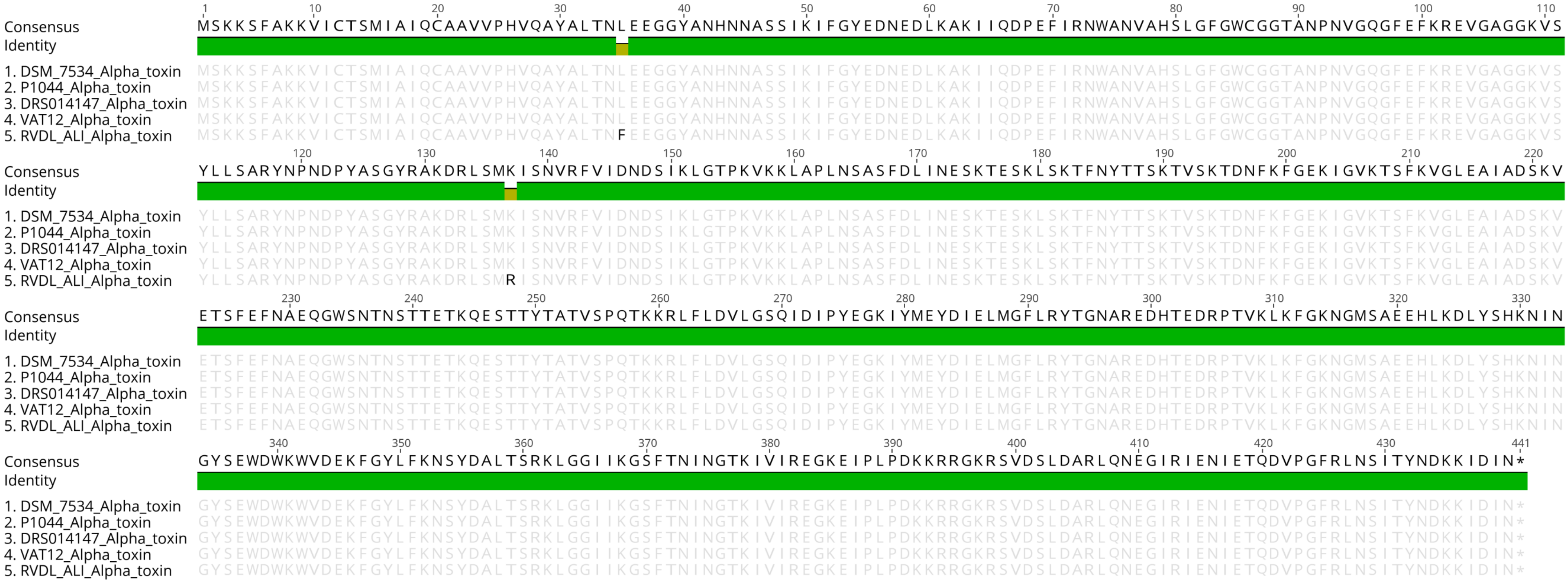
Consensus
Identity
1. DSM_7534_Alpha_toxin
2. P1044_Alpha_toxin
3. DRS014147_Alpha_toxin
4. VAT12_Alpha_toxin
5. RVDL_ALI_Alpha_toxin
MSKKSFAKKVICTSMIAIQCAAVVPHVQAYALTNLEEGGYANHNNASSIKIFGYEDNEDLKAKIIQDPEFIRNWANVAHSLGFGWCGGTANPNVGQGFEFKREVGAGGKVS
F
YLLSARYNPNDPYASGYRAKDRLSMKISNVRFVIDNDSIKLGTPKVKKLAPLNSASFDLINESKTESKLSKTFNYTTSKTVSKTDNFKFGEKIGVKTSFKVGLEAIADSKV
R
ETSFEFNAEQGWSNTNSTTETKQESTTYTATVSPQTKKRLFLDVLGSQIDIPYEGKIYMEYDIELMGFLRYTGNAREDHTEDRPTVKLKFGKNGMSAEEHLKDLYSHKNIN
GYSEWDWKWVDEKFGYLFKNSYDALTSRKLGGIIKGSFTNINGTKIVIREGKEIPLPDKKRRGKRSVDSLDARLQNEGIRIENIETQDVPGFRLNSITYNDKKIDIN*

B

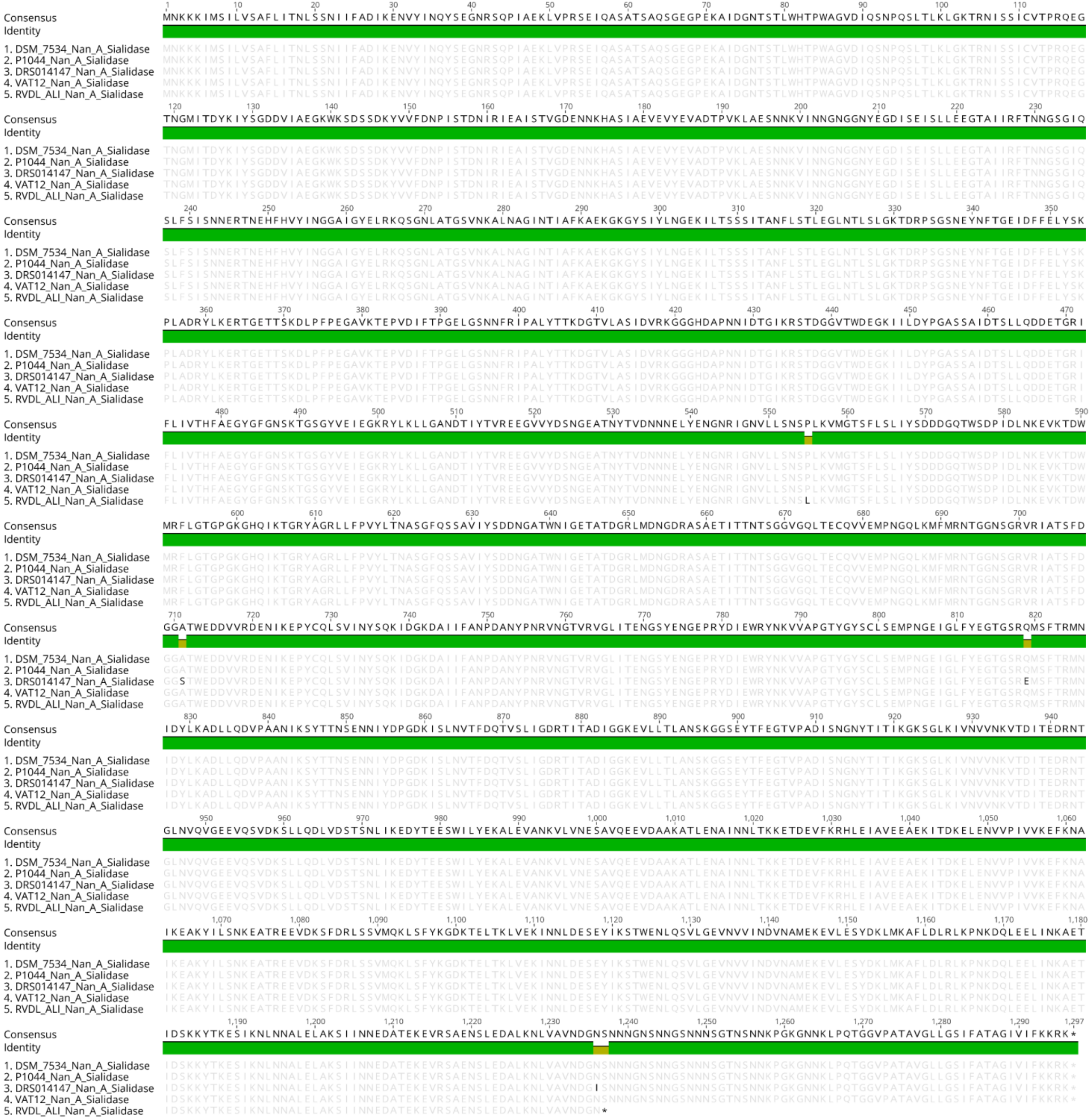
Consensus
Identity
1. DSM_7534_Nan_A_Sialidase
2. P1044_Nan_A_Sialidase
3. DRS014147_Nan_A_Sialidase
4. VAT12_Nan_A_Sialidase
5. RVDL_ALI_Nan_A_Sialidase

C

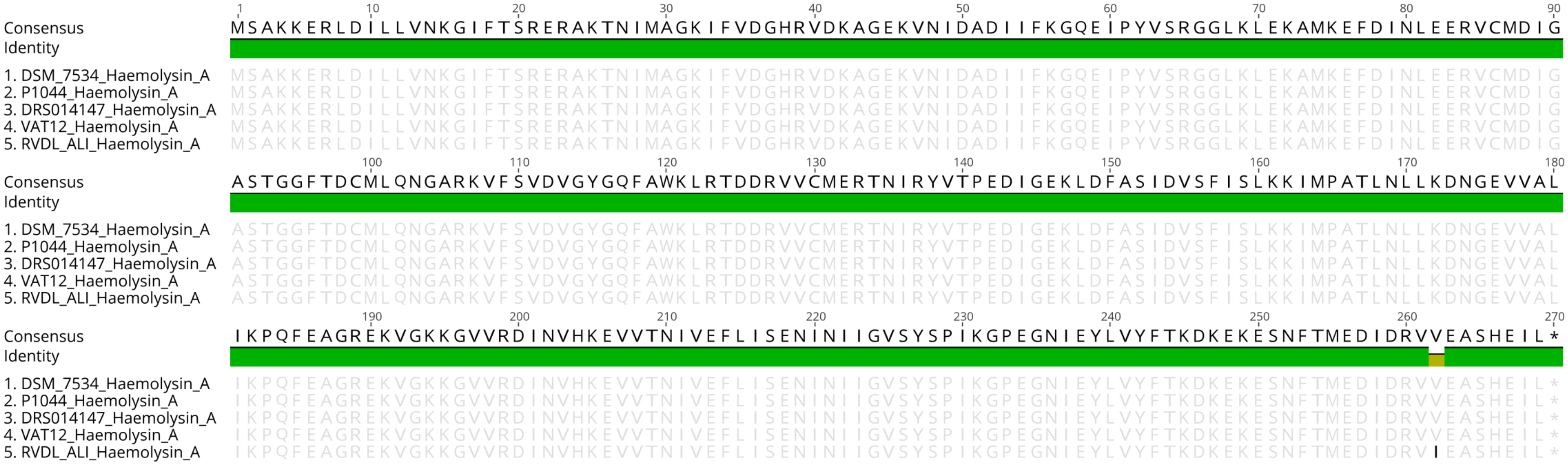

Consensus
Identity
1. DSM_7534_Haemolysin_A
2. P1044_Haemolysin_A
3. DRS014147_Haemolysin_A
4. VAT12_Haemolysin_A
5. RVDL_ALI_Haemolysin_A
MSAKKERLDILLVNKGIFTSRERAKTNIMAGKIFVDGHRVDKAGEKVNIDADIIFKGQEIPYVSRGGLKLEKAMKEFDINLEERVCMDIG
ASTGGFTDCMLQNGARKVFSVDVGYGQFAWKLRTDDRVVCMERTNIRYVTPEDIGEKLDFASIDVSFISLKKIMPATLNLLKDNGEVVAL
IKPQFEAGREKVGKKGVVRDINVHKEVVTNIVEFLISENINIIGVSYSPIKGPEGNIEYLVYFTKDKEKESNFTMEDIDRVVEASHEIL*

D

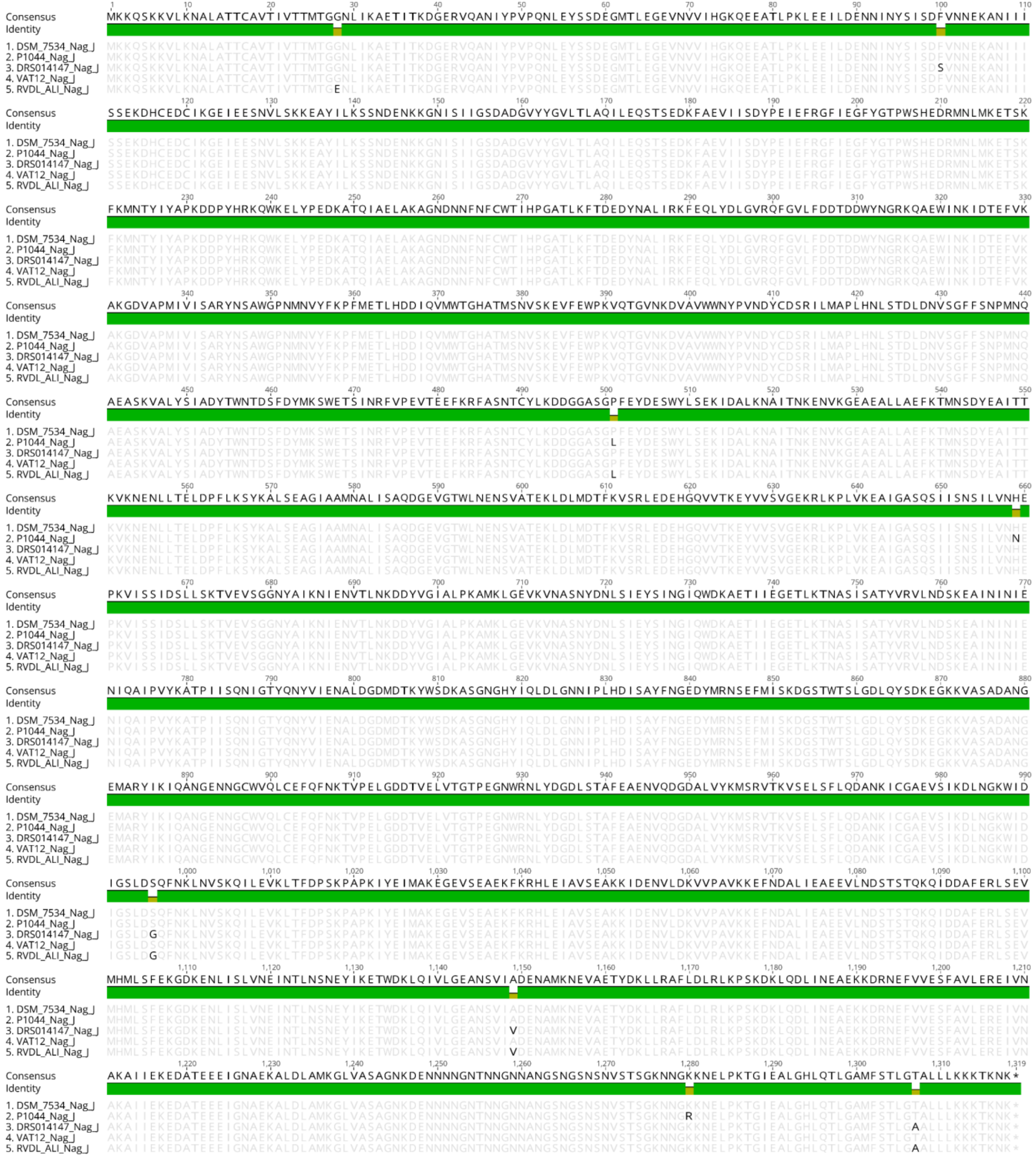

Consensus
Identity
1. DSM_7534_Nag_J
2. P1044_Nag_J
3. DRS014147_Nag_J
4. VAT12_Nag_J
5. RVDL_ALI_Nag_J

E

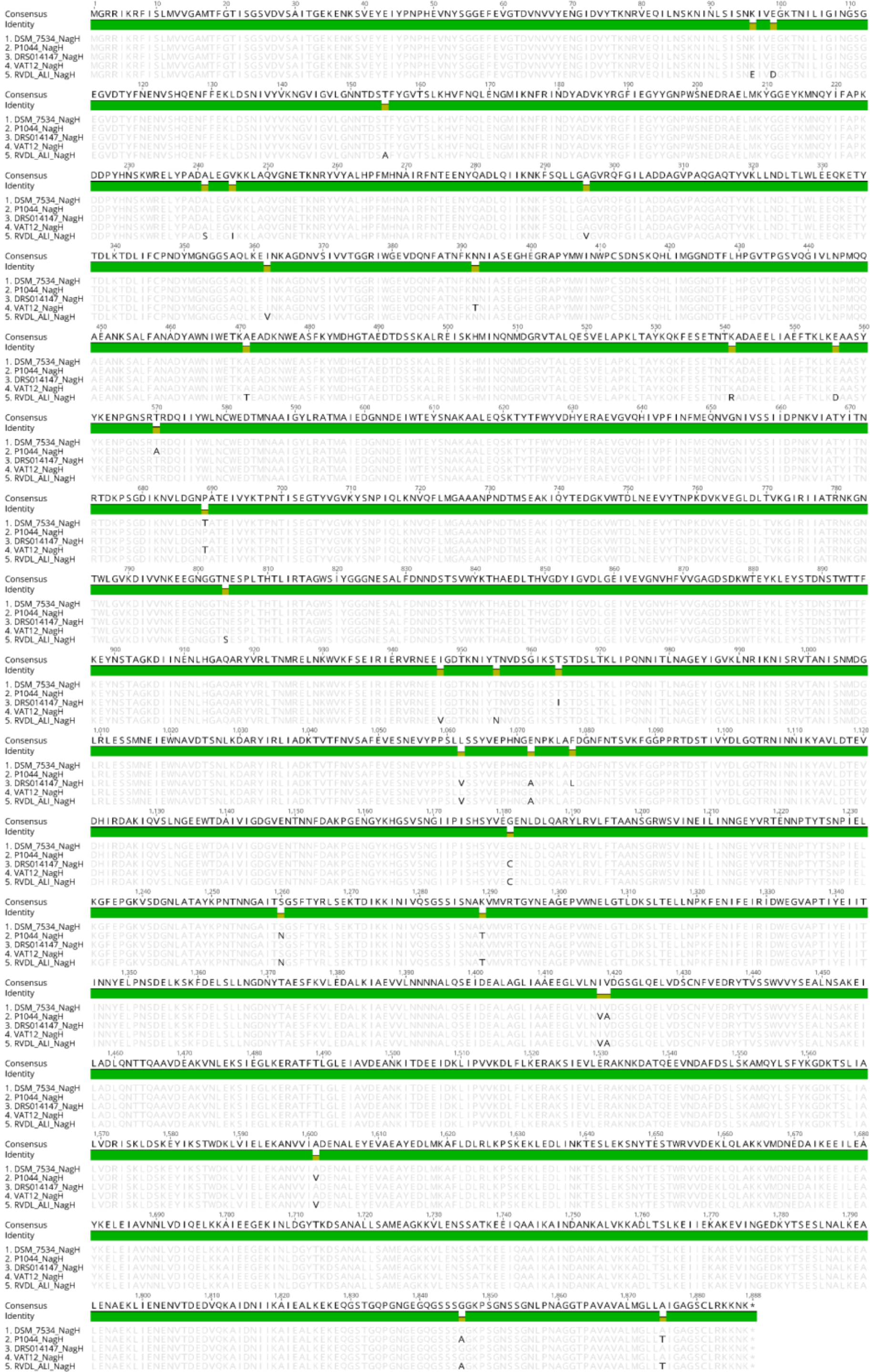
Consensus
Identity
1. DSM_7534_NagH
2. P1044_NagH
3. DRS014147_NagH
4. VAT12_NagH
5. RVDL_ALI_NagH
